# Consumption of Common Bean Suppresses the Obesogenic Increase in Adipose Depot Mass: Impact of Dose and Biological Sex

**DOI:** 10.3390/nu15092015

**Published:** 2023-04-22

**Authors:** Henry J. Thompson, Tymofiy Lutsiv, John N. McGinley, Vanessa K. Fitzgerald, Elizabeth S. Neil

**Affiliations:** Cancer Prevention Laboratory, Colorado State University, Fort Collins, CO 80523, USA

**Keywords:** adipose tissue, anti-obesogenic activity, obesity prevention, biological sex effects, common bean, pulses, immune function, lipid metabolism, precision nutrition

## Abstract

Obesity prevention is stated as a simple objective in the public health guidelines of most countries: avoid adult weight gain. However, the success of the global population in accomplishing this goal is limited as reflected in the persisting pandemic of overweight and obesity. While many intervention strategies have been proposed, most are directed at mitigating the consequences of obesity. Efforts intended to prevent unintentional weight gain and associated adiposity are termed anti-obesogenic. Herein, evidence is presented that a neglected category of foods, pulses, i.e., grain legumes, have anti-obesogenic activity. Using a preclinical mouse model of obesity, a dose–response study design in animals of both biological sexes, and cooked, freeze-dried, and milled common bean as a representative pulse, data are presented showing that the rate of body weight gain is slowed, and fat accumulation is suppressed when 70% of the dietary protein is provided from common bean. These anti-obesogenic effects are reduced at lower amounts of common bean (17.5% or 35%). The anti-obesogenic responsiveness is greater in female than in male mice. RNA sequence analysis indicates that the sex-related differences extend to gene expression patterns, particularly those related to immune regulation within adipose tissue. In addition, our findings indicate the potential value of a precision nutrition approach for human intervention studies that identify “pulse anti-obesogenic responders”. A precision approach may reduce the concentration of pulses required in the diet for benefits, but candidate biomarkers of responsivity to pulse consumption remain to be determined.

## 1. Introduction

For decades, one of the most consistent public health guidelines to promote human well-being and reduce chronic disease has been to avoid unintended adult weight gain [1]. The statement of this universal guideline in this manner recognizes the value of the intentional increase in muscle mass and that the goal of weight gain avoidance is targeted to fat mass and ectopic lipid deposition. To achieve this objective, there has been an appropriate focus on maintaining levels of physical activity above those associated with a sedentary lifestyle and on limiting caloric intake to a level that provides the recommended intake of dietary nutrients [2]. Collectively, maintaining a physically active lifestyle and limiting excessive caloric intake create an energy balance equation consistent with the avoidance of excessive fat store accumulation and associated chronic diseases. However, based on trends in overweight and obese individuals, the majority of the global population in both developed and developing countries are failing to comply with the goal of avoiding unintended adult weight gain [3].

The unabated pandemic of obesity affects the physical, mental, social, and financial wellness of individuals, the communities in which they live, and the healthcare systems that manage their medical care. Consequently, a range of solutions are being marketed to the public which include drugs, nutraceuticals, surgical procedures, foods, and food patterns [4]. This constitutes the weight control industry valued at $175.94 billion in 2017 in the U.S.A, which is projected to reach $303.81 billion by 2027 at a compound annual growth rate (CAGR) of 6.9% [5]. The size of this market has several implications that include: (1) there is widespread interest in weight control, although this is primarily focused on weight loss rather than avoidance of weight gain, (2) the long-term success rate of the above-described interventions is low, and (3) many of the available interventions fail either because they are not scientifically credible, or because they are being used with the mentality that *one size fits all* rather than with the precision approaches in nutrition and in public health interventions that have become mainstream over the last twenty years [6,7].

A focus of nutritional epidemiologists investigating chronic disease risk over the last decade has been identifying contrasting food patterns in various populations associated with low or high disease risk [8]. Of the many important observations that have emerged from that work, considerable evidence supports the value of whole-food-based diets and the detrimental effects of dietary patterns high in ingredient-based foods and, particularly, in the content of ultra-processed foods [9,10]. With regard to which types of whole foods are beneficial, the topic must be approached with caution because taking a specific food type out of the pattern, in which it is typically consumed, may alter its biological impact. Mindful of this concern, our laboratory has sought to determine the effects of a specific and under-consumed food category—pulses, i.e., grain legumes [11]. This focus was prompted by a series of population studies and their systematic review indicating that pulse consumers have modest improvements in weight control and chronic disease risk relative to non-pulse consumers [12,13]. In a series of experiments, we have used two preclinical models of obesity in rats and mice to evaluate the biological plausibility of the epidemiological evidence and have demonstrated pulse-specific anti-obesogenic activity, i.e., a specific pulse—common bean—suppressed the excessive accumulation of body fat using the gold standard preclinical approach of paired feeding [14,15]. In those studies, the control and bean-fed rats and mice had the same final body weights at study termination, but the bean-fed animals had statistically significant reductions in body fat. That work was done using diet formulations in which the common bean provided 70% of the dietary protein. This level of intake is undoubtedly feasible in human populations, given that vegan food patterns exist and are gaining in popularity. However, we judge that what is needed is an investigation of the dietary concentration of a pulse, such as the common bean, that can sustain benefits and whether the biological sex of the animal impacts the dose threshold at which anti-obesogenic activity occurs.

The research objectives of this project are: (1) to determine the minimum dietary quantity of the common bean, expressed as a percentage of the total dietary protein, that suppresses body weight gain, mainly via the reduced accumulation of fat in the subcutaneous and visceral adipose tissues; (2) to determine whether biological sex affects the amount of the common bean required to modulate these outcomes, (3) to use immune nanocapillary electrophoretic quantification of key proteins to probe bean-mediated effects on lipid metabolism, and (4) to explore gene expression profiles using RNA sequence analysis to identify biological functions modulated by the anti-obesogenic activity of the common bean. 

## 2. Materials and Methods

The animal feeding study from which the anthropometric data and the tissue analyses described herein were obtained has been reported [16]. Accordingly, the experimental design is briefly summarized, while the laboratory analyses specific to this study are presented in detail.

### 2.1. Animal Feeding Study

Male and female C57BL6/J mice from the Jackson Laboratory (Barr Harbor, ME, USA) were group-housed using standard husbandry conditions including *ad libitum* access to food and water and a 12-h light/dark cycle. The room temperature was maintained at 27.5 ± 2 °C. Until eight weeks of age, animals were fed a purified diet containing 32.5% kcal. Then, each experimental diet group included 20 animals per each biological sex using staggered randomization according to their body weights. The experimental duration was 12 weeks in female mice and 14 weeks in male mice, which necessitated maintaining the quality of necropsy procedures and the organization of tissue labeling and storage following each necropsy. Mice euthanasia included isoflurane-induced anesthesia followed by cervical dislocation. Subcutaneous and visceral fat pads were excised, weighed, and then snap-frozen in liquid nitrogen and stored at −70 °C. All the procedures performed on animals complied with the Colorado State University Institutional Animal Care and Use Committee guidelines (protocol KP 1431). 

### 2.2. Experimental Diets

Diet formulations were designed to provide equal total caloric energy, as reported in [16]. For convenience, their formulation was included in Supplemental Table S1. Briefly, bean doses were designated as 0% (bean-free control), 17.5%, 35%, and 70% of the total dietary protein derived from beans. The total dietary fiber concentration of beans was 23.5 g/100 g bean powder [17]. A low-fat isocaloric diet formulation was used as a negative control as it contained only 11% dietary calories from fat.

### 2.3. Histopathology 

A piece of frozen subcutaneous or mesenteric fat (*n* = 4 per group) was placed in specimen-embedding media (Sakura Finetek, Torrance, CA, USA) on an object disk and frozen in a cryochamber (Leica Biosystems, Deer Park, IL, USA). Ten-micron sections of tissue were placed on charged slides (Statlab Medical Products, McKinney, TX, USA), and stored at −70 °C.

#### 2.3.1. Fixation

Slides of frozen, sectioned adipose tissue were taken out of the freezer, inserted in a slide carrier, and placed on the bench to thaw for 5 min before fixing in 10% buffered formalin. 

#### 2.3.2. Hematoxylin and Eosin (H&E) Staining

Tissue sections were washed in distilled water and stained using hematoxylin and eosin. Sections were dehydrated using an increasing stepwise percentage of ethyl alcohol (95% × 2, 100% × 2, and isopropanol × 1) and xylene prior to coverslipping with synthetic resin. 

### 2.4. Western Blot-Based Nanocapillary Electrophoresis

Protein lysates were prepared as previously described [14]. The Bradford assay was used to ascertain the concentration of protein. 

The Jess instrument and paired reagent kits were used for conducting electrophoresis in proprietary nanocapillary cartridges (ProteinSimple, San Jose, CA, USA). Kits included specialized plates (containing matrix and running buffer, a 25-capillary cartridge, fluorescent standards, and ladder), 10X sample buffer, Horseradish peroxidase (HRP)-labeled secondary antibody, Near-infrared (NIR)-labeled streptavidin for the ladder, peroxide and luminol-S, milk-free antibody diluent, and wash buffer. Samples were processed by combining the necessary volume of protein lysate with 1.5 µL of 5X fluorescent master mix and 0.1X sample buffer in a 0.2 mL PCR tube with an ultimate concentration of 0.2 mg/mL. A dry bath set at 95°C was used to denature the samples for 5 min. Samples were vortexed, centrifuged briefly, and kept on ice prior to loading the plate. Samples, biotinylated ladder, protein normalization reagent, primary antibodies, HRP-conjugated secondary (samples only), NIR-labeled secondary antibody (ladder only), and chemiluminescent substrate (luminol-S mixed with peroxide) were loaded to the plate. The protein normalization reagent stock solution was reconstituted and diluted to a working concentration according to the specific instructions for each kit (12–230 kDa or 66–440 kDa). Wash buffer was added before centrifuging the plate at 1000× *g* for 5 min. 

The components (plate and capillary cartridge) were placed in the automated Jess instrument. 375 volts (12–230 kDa plate) or 475 volts (66–440 kDa plate) were applied for 30 min to separate proteins in the capillary based on their molecular weight. Proteins in the capillaries were immobilized using UV light prior to the addition of the protein normalization reagent for total protein determination via NIR. The next steps included blocking with milk-free antibody diluent, labeling with antibodies, and signal detection. The resulting chemiluminescent signal of protein targets and a fluorescent signal of the total protein were displayed as an electropherogram and rendered as an image similar to a traditional western blot. Data were normalized by dividing the peak area of the target protein by the total protein area of the sample within each capillary normalized per total protein area across all the capillaries in the plate per each run for batch effects. 

### 2.5. RNA Isolation and RNA-Seq Analysis

Two groups of bean diets—0% and 70%—were chosen for RNA sequencing (RNA-Seq) analysis. Frozen adipose tissue samples (*n* = 20 per group) were ground in ceramic mortars using ceramic pestles pre-chilled with liquid nitrogen. During this process, samples were pooled (2 samples per pool) according to a similar pattern of weight gain, resulting in total *n* = 10 pools/group. RNA samples were extracted, sequenced, and analyzed, as reported in [16]. The gene expression data contained 25,683 annotated genes, out of which 21,859 were protein-coding. Differentially expressed genes (DEGs) were obtained in the QIAGEN CLC Genomics Workbench, version 23.0.2, using Differential Expression for RNA-Seq workflow provided with the software. Obtained *p*-values, *p*-values corrected for multiple comparisons using Benjamini–Hochberg procedure controlling the false discovery rate (FDR; *q*-values), expression fold change, expression log_2_ ratios, and the expression intensities (the mean maximum of the transcripts per million (TPM) per diet group in a comparison pair) were exported and uploaded to the QIAGEN Ingenuity Pathway Analysis (IPA) software, v81348237 (QIAGEN, Redwood City, CA, USA), https://digitalinsights.qiagen.com/IPA (accessed on 13 January 2023) [18], for further analysis. 

Within IPA, datasets were subjected to quality filtering to obtain only data on DEGs with the expression intensity > 10 TPM, *p*-value < 0.05, and log_2_ ratio > |0.6| to keep each comparison pair dataset within recommended by IPA limits (100–3000 analysis-ready molecules). We performed a Core Analysis using the expression log_2_ ratio as a measurement to calculate directionality in the analysis. The QIAGEN Ingenuity Knowledge Base was used to evaluate the results. Graphical Summaries were constructed according to heuristic IPA algorithms. Briefly, the top-most significant entities from the results of Core Analysis engines are selected into a network: from the Canonical Pathways (by *p*-value ≤ 0.05 of overlap), from Upstream Analysis (by the magnitude of observed differential expression of DEGs (by log_2_ ratio) that matched significant genes, mRNAs, and proteins with significant *p*-values (≤0.05) and *z*-scores (≥|2|), and from Diseases & Functions with *p*-values ≤ 0.05 and *z*-scores ≥ |2|. In addition to relationships between the selected entities from the results of Core Analysis, a Graphical Summary was included. The latter additionally contains inferred relationships (such as Inferred Causation (IC) in presented figures herein by the dotted lines) built using IPA’s machine-learning models based on the normalized and weighted values from curated findings from the QIAGEN Ingenuity Knowledge Base and the patterns of observed DEGs (log_2_ ratio) and predicted by the analyses relationships (*z*-score), providing new connections between entities that are not used elsewhere in IPA. As a result, only the most significant entities from RNA-Seq analysis that connect to one another comprise the Graphical Summary. 

### 2.6. Statistical Evaluation

In general, anthropometric, protein, and gene expression data were subjected to factorial analysis of variance (ANOVA) with the diet group, tissue type, and sex as the factors. Depending on the data distribution (D’Agostino & Pearson omnibus normality test), some data were evaluated via the Wilcoxon signed-rank test or the Kruskal–Wallis rank order test. These details are noted in the data presentation. For pairwise comparisons, the Tukey or Dunn’s *post hoc* tests were used. Benjamini-Hochberg method was used for multiple testing correction of *p*-values. Differences were considered significant with *p* < 0.05. Data analyses were conducted using SYSTAT, version 13.0; SAS, version 9.2; GraphPad Prism, version 5.2; or *R*, version 4.2.2 using *FSA*, *rstatix*, and *gpubr* packages. 

## 3. Results

### 3.1. Body Weight Gain

**Figure 1:**
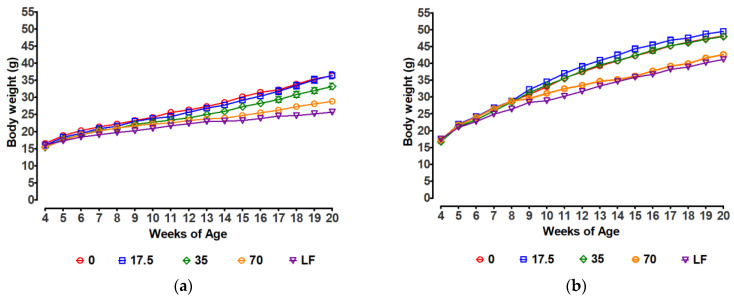
Effect of the common bean on body weight gain in mice. Female (**a**) and male (**b**) mice were fed a diet formulation containing 32.2% dietary calories from fat and 0% to 70% of dietary protein from common bean. Low-fat diet (11% dietary calories from fat, LF) was used as a negative control. Values are means ± SEM; *n* = 20 mice per data point. Note that most SEM were within the range of the symbol size.

The standard purified diet fed to laboratory mice provides 11% of calories from fat. In the C57BL6/J model of dietary-induced obesity, this low-fat diet formulation serves as a negative control, i.e., it is generally recognized as being anti-obesogenic. C57BL6/J mice are known to accelerate weight gain and fat accumulation, as the percent of dietary calories from fat is increased from 11% to 60%. However, 60% of dietary calories from fat are not considered physiologic [19,20]. In this study, the “obesogenic tone” of the diet was set at 32.2% of dietary calories from fat since it is obesogenic in C57BL6/J mice and also is the recommended level of dietary fat for people [2]. When common bean provided 0%, 17.5%, 35%, or 70% of the dietary protein in the 32.2% dietary fat formulation, the change in body weight gain over time for female and male mice is shown in Figure 1. The growth rate for the low-fat diet is also shown. The divergence in body weights among the treatment groups was gradual, becoming apparent after 4 weeks on diet in female mice and at 2 weeks after experimental diet feeding was initiated (week 10 of age) in male mice. A dose-dependent reduction in the body rate gain was observed in female mice, whereas the only dose of the common bean that affected growth in the male mice was 70% of protein from bean. In the female mice, the bean effect threshold was 35%, noting that the rate of body weight gain in the 17.5% group was indistinguishable from the high-fat (0%) control group. In the male mice, the bean effect threshold was 70%. These data support the premise that each biological sex exhibits differential responsivity to common bean.

### 3.2. Anthropometric Data at the End of the Study

Table 1 shows the tabulation of the endpoint anthropometric data. The final body weight statistics indicate that the threshold for differences was 35% of the dietary protein from bean in females but 70% in males. It is common in this model to normalize body weight and organ mass to the tibia length. When this is done for body weight, the measurement corresponds to the body mass index (BMI) in humans, and therefore, it is referred to as such herein. From these data, three points can be made: (1) both female and male mice fed the 32.2% kcal from fat had excess body mass relative to the tibia length in comparison to the low-fat control, (2) females had a lower BMI than males in both the high- and low-fat control groups, and (3) the pattern of BMI reduction was consistent with that gleaned from the final body weight data. Of greater insight was the percent body fat data. Note that the total body fat is the amount excised from specific fat depots and is not all body fat. Nonetheless, these data illustrate a marked reduction in body fat relative to body weight at 35% and 70% of the dietary protein from bean in female mice and at 70% in male mice. This finding is consistent with specific effects of bean on fat accumulation that exceed those generally correlated with differences in body weight. Values for subcutaneous and visceral fat normalized to the tibia length are also reported. The reductions were significant at the 70% level in both depots in females, but only the subcutaneous fat pad was reduced in males. 

Visceral fat is stored in several depots, as described in [21]. Of these, three were measured in this study and reported in Table 2. They were selected partly because of their metabolic importance (mesenteric) and partly because they are commonly measured in the C57BL6/J model system (retroperitoneal and perigonadal), even though there is no equivalent to an epididymal fat pad in male humans. Mesenteric fat was reduced in both female and male mice at 70% of the dietary protein from bean. Similarly, both parametrial and retroperitoneal fats were reduced in female mice. However, retroperitoneal fat was refractory to change, while epididymal fat was actually higher in male mice at 70% of the dietary protein from bean and also in the low-fat control group. These differences explain the lack of overall reduction in visceral fat in male mice reported in Table 1.

**Figure 2:**
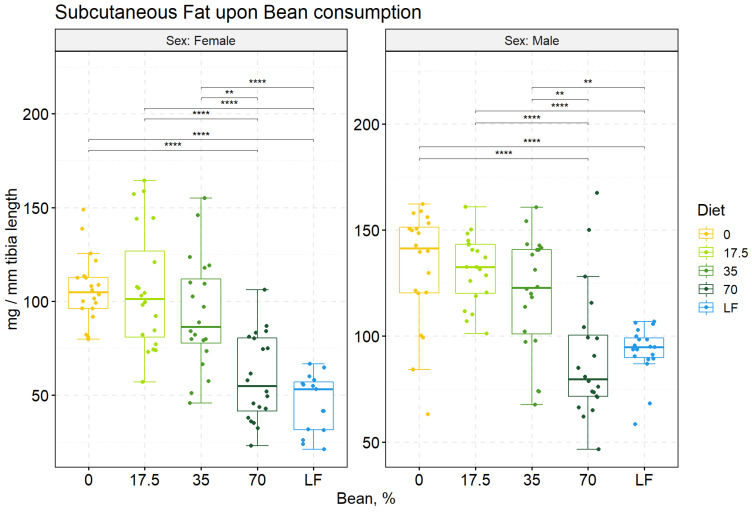
Subcutaneous adipose tissue weight changes upon the consumption of the common bean in female and male mice. Tissue weights were normalized by the mm of the tibia length. Bean-free (0%) and low-fat diets were used as positive and negative controls, respectively. One-way ANOVA test indicated significant differences (*F* = 22.378, *p*-value = 7.531 × 10^−13^) in the female cohort (**left panel**). Pairwise comparisons between the diet groups were conducted using the Tukey test. Male cohort (**right panel**) data were analyzed using the Kruskal–Wallis test with χ^2^ = 39.051, *p*-value = 6.798 × 10^−8^ with the *post-hoc* Dunn testing for pairwise comparisons. The Benjamini-Hochberg procedure was used for the multiple testing correction of *p*-values. ** *p*-value < 0.01; **** *p*-value < 0.0001.

**Figure 3:**
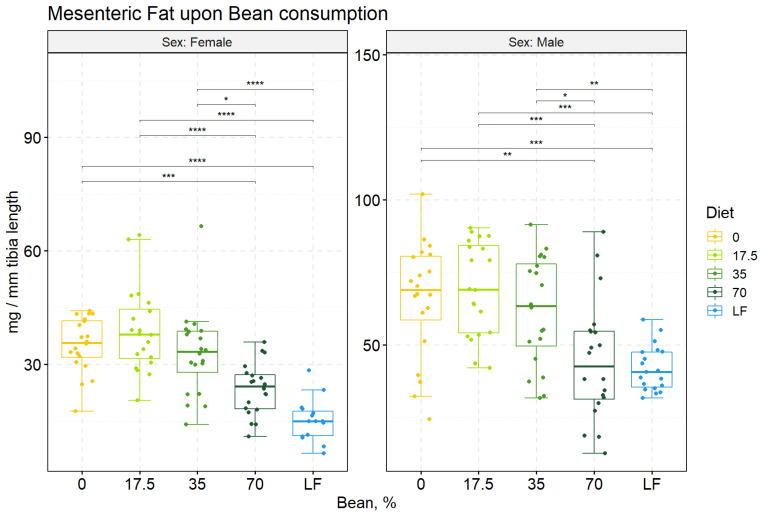
Mesenteric adipose tissue weight changes upon the consumption of the common bean in female and male mice. Tissue weights were normalized by the mm of the tibia length. Bean-free (0%) and low-fat diets were used as positive and negative controls, respectively. The Kruskal-Wallis test indicated χ^2^ = 48.776, *p*-value = 6.502 × 10^−10^ in the female cohort (**left panel**) and χ^2^ = 30.467, *p*-value = 3.931 × 10^-6^ in the male cohort (**right panel**). Pairwise comparisons between the diet groups were conducted using the *post-hoc* Dunn test with the Benjamini-Hochberg procedure for the multiple testing correction of *p*-values. * *p*-value < 0.05; ** *p*-value < 0.01; *** *p*-value < 0.001; **** *p*-value < 0.0001.

Subcutaneous adipose tissue plays a significant role in thermoregulation, whereas the visceral fat pads, in general, and the mesenteric fat pad, in particular, maintain metabolic homeostasis during feeding/fasting cycles [21]. During the statistical analysis of the subcutaneous and mesenteric fat depot data, a wide range in the minimum and maximum data points was observed. That prompted us to visualize that data using box plots (Figure 2 and Figure 3). Because of the data range, which was particularly prominent in male mice fed 70% of the dietary protein from bean, we speculated that a median split of the mice (male or female) into subgroups within each treatment group would result in statistically significant subgroups, and this suspicion was confirmed in the 0% and 70% diet groups. 

Histological sections of both subcutaneous and mesenteric fat depots were evaluated in female and male mice fed 0% or 70% of the dietary protein from bean (Supplemental Figure S1). No remarkable differences in the connective tissue stroma were observed between diet groups, consistent with the differences in adipose depot mass, reflecting overall differences in fat accumulation. Fat cell size and shape were heterogeneous within and between the diet groups, indicating the value of determining tissue mass rather than using morphometric analysis of histological sections that would require census counting of large fields of cells. Morphometric analysis was not possible because most of the tissue was used for either RNA or protein isolation.

### 3.3. Mechanisms

Because of the importance of subcutaneous (thermoregulatory homeostasis) and mesenteric (metabolic homeostasis) fat in energy metabolism and the fact that feeding common bean suppressed fat accumulation in both depots in female and male mice, these tissues were the focus of our analyses. The experimental approach had two components. The hypothesis-driven analyses focused on the regulation of fatty acid metabolism using gene expression data from the RNA-Seq analysis and protein expression data determined with Western blot analysis using immune nanocapillary electrophoresis. The data-driven approach used the RNA-Seq analysis coupled with bioinformatic analyses using the QIAGEN Ingenuity Pathway Analysis (IPA) software.

#### 3.3.1. Hypothesis-Driven Analyses

There is a strong literature basis for presuming that lipid metabolism will be affected in adipose depots under anti-obesogenic conditions. The initial bioinformatic evaluation of the RNA-Seq data at the level of canonical pathway involvement did not identify lipid metabolism among the pathways of the greatest prominence. Therefore, we limited our query to three gene/protein targets generally reported: peroxisome proliferator-activated receptor γ (PPARγ), considered a master regulator of adipogenesis and lipid metabolism, fatty acid synthase (FASN), and stearoyl-Coenzyme A desaturase (SCD). The expression data for these genes are provided in the Supplementary Figure S2. The protein expression data are shown in the Table 3, with the actual Western blots provided in the Supplementary Figure S3. No evidence was found to support the role of these proteins in accounting for the effect of common bean.

#### 3.3.2. Data-Driven Analyses

**Figure 4:**
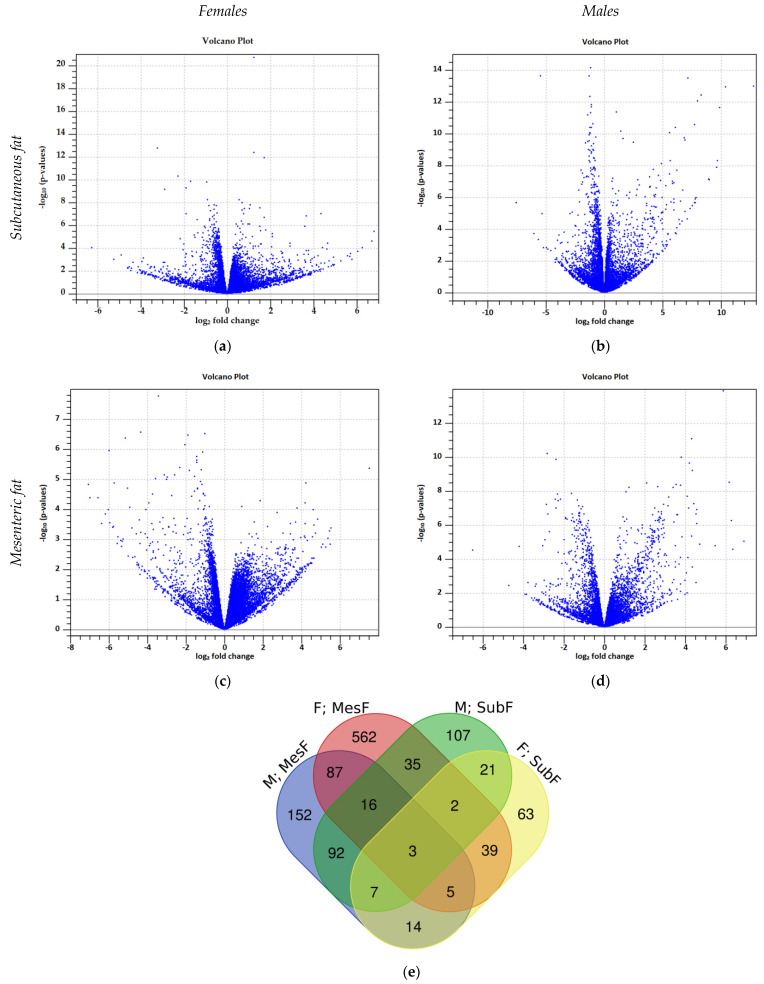
Distribution of differentially expressed genes (DEGs) in 70% versus 0% bean dose diets across sex and tissue cohorts. (**a**–**d**) Volcano plots of raw DEGs depicting significance (−log_10_ *p*-values) and effect size (log_2_ fold change) of DEGs upon bean consumption from the QIAGEN CLC Genomics Workbench. (**e**) Comparative Venn diagram of analysis-ready DEGs subjected for analysis at QIAGEN IPA.

To determine the molecular signature of dietary bean effects, we focused only on 0% and 70% bean-containing diets in both sex cohorts. Gene expression analysis demonstrated a clear separation of bean-fed from bean-free diet groups in both female and male cohorts according to volcano plot analysis (Figure 4a–d). After applying quality filters, there were 749 differentially expressed genes (DEGs) in the female cohort versus 376 DEGs in the male cohort of mesenteric fat samples. In contrast, subcutaneous fat tissue contained only 154 DEGs versus 283 DEGs in the female and male cohorts, respectively (Figure 4e). 

To objectively determine the most distinct DEGs affected by the consumption of bean at the 70% of the total dietary protein content, we ranked them by the largest absolute values of log_2_ fold change and by the smallest FDR-corrected *p*-values and averaged these ranks to obtain a combined rank. Among the unique DEGs for female subcutaneous fat, there was bean-increased expression of *Dmbt1*, *Dbp*, *Mycl*, *Prtn3*, *Syn2*, *Pcp4*, *Ptn*, *Mpzl2* and decreased expression of *Otop1*, *Cela1*, *Rnase2*, *H3c8*, *Scd2*, *Mogat2*, *Kcnj14*. In turn, males exhibited most significant changes in elevated *Amy2a*, *Fasn*, *Tst*, *Alox12e* and downregulated *Pik3cd*, *Cyp4f2*, *Lcn2*, *Sash3*, *Daglb*, *Cxcl13*, *Hla-Dmb*, *Timp1*, *Atf3*, *Il10ra*, *Clec4a3* in subcutaneous fat. Higher levels of *Dapl1*, *Myh1*, *Krt15*, *Lgals7*/*Lgals7b*, *Krt27*, *Tchh*, *Krt71*, *Krt25*, *Cyp2f1*, *Krtap7-1*, *Krtap8-1*, *Krt73* as well as lower expression of *Tph2* and *Gpnmb* signified bean effects among the shared DEGs between females and males in subcutaneous fat. Among them, *Cox6a2* was upregulated in females but downregulated in males. Similar opposite patterns exhibited *Dpep2*, *Myoc*, *Eno3*, and *Des* being upregulated in females but decreased in males, whereas *Fabp2* and *Timp4* were reduced in females but elevated in males upon the influence of dietary bean. 

In mesenteric fat, downregulated *Pla2g2e*, *Hephl1*, *Peg10*, *Prg4*, *Mogat2*, *Alb*, *Lep*, *Folh1*, *Ctrl*, *Cela2a*, *Acta1*, *Apoa1*, *Scd2* and upregulated *2210010c04rik*, *Rnase1*, *Ctrl*, *Cela2a* were the top 15 distinct DEGs of females that were unshared with males. For male counterparts, bean-increased *Il22ra2*, *Marco*, *Cr2*, *Fcer2*, *Pax5*, *Cd19*, *Cxcr5*, *Dnase1l3*, *Blk*, *Tcf7*, *Timd4* and bean-decreased *Mmp12*, *Itgad*, *Rnf128*, *Atp6v0d2* were the most significant unique DEGs. The top 15 most distinct and shared between the sex cohort DEGs in mesenteric fat were *Glycam1*, *Apol7a*, *Cd209b*, *Ccl21*, *Lef1*, *Cd5l*, *Dtx1*, *Ccl19*, *Lat*, *Cd8a*, *Madcam1*, *Carmil2*, *Il4i1*, *Dennd2d*, *Ctrb2*. All of them were upregulated upon bean consumption, except for the chymotrypsinogen B2 (*Ctrb2*), whose levels were increased in females but reduced in males. Analogously, *Adam8*, *Ubd*, *Rgs1*, *Glipr1*, *Ifi16*, *Slc15a3*, *Kcnn4*, *Hmga1*, and *Slamf8* were elevated in females but suppressed in males, in contrast to *Timp4*, *Ptges*, *Agt*, *Thbd*, *Morc4*, whose levels were increased in males but reduced in females upon consuming beans. 

The top bean-induced DEGs in females shared by both adipose depots are suppressed *Mogat2*, *Scd2*, *Pla2g2e*, *Paqr9*, *Lep*, *Serpina3*, *Igfals*, *Fgf13*, *Pcolce2*, *Timp4*, *Sfrp5*, *Syt12*, *Saa3*, *Lbp*, and enhanced *Dbp.* Also in that list, increased expression of *Ccl21*, *Cd79a*, *Ubd* and reduced *Agt* in the mesenteric fat, which had the opposite expression changes upon consumption of beans in the subcutaneous depot of females. Amongst the common DEGs for two depots in males, the most distinct were *Atp6v0d2*, *Mmp12*, *Slc37a2*, *Trem2*, *Hpgds*, *Gpnmb*, *Cd300ld*, *Itgad*, *Cd68*, *Cd300a*, *Hk3*, *Adgre1*, *Clec12a*, *Cd84*, *Gpr50*, all of which were suppressed upon bean consumption. In turn, *Cd3e*, *Epsti1*, *Hla-Dmb*, *Ccl5*, *Gimaps*, *Ms4a6b*, *Cxcl13*, and *Cd37* were all elevated in the mesenteric fat but suppressed in the subcutaneous fat in males. Only three DEGs were shared across both tissue and sex cohorts: suppressed serum amyloid A 3 (*Saa3*), reduced in females but increased in males tissue inhibitor of metalloproteinases 4 (*Timp4*), and ubiquitin D (*Ubd*), which was downregulated in subcutaneous fat but upregulated in the mesenteric fat of female mice, yet suppressed in both fat pads of males. 

**Figure 5:**
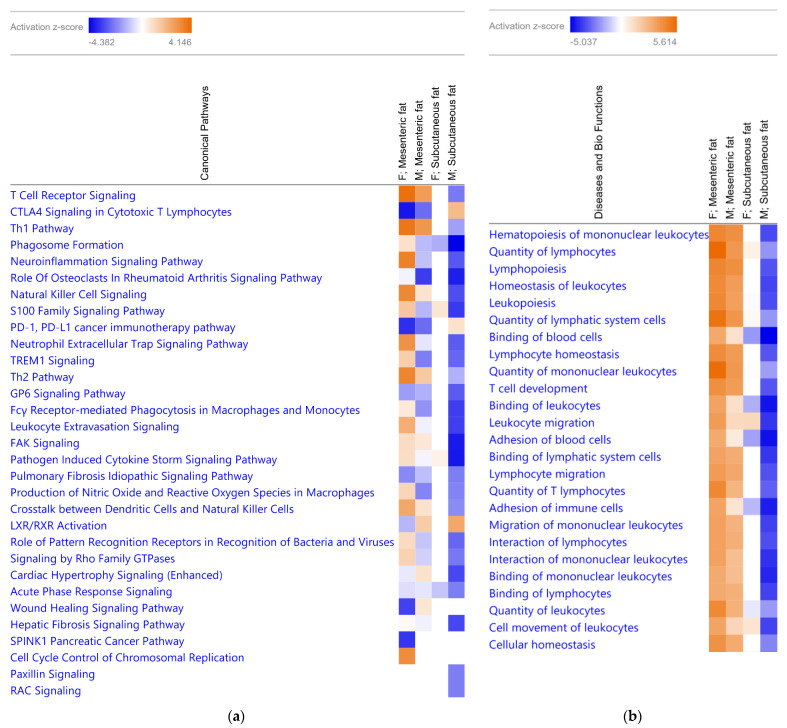
Functional annotation of bean-induced DEGs in subcutaneous and mesenteric fact of female and male mice from QIAGEN IPA Core analysis: (**a**) Canonical pathways analysis results organized by their *z*-scores across the cohorts; (**b**) Top 25 most significantly activated or inhibited diseases and biological functions assigned to bean-induced DEGs. Heatmap denotes activation (orange) or inhibition (blue) of respective functions based on activation *z*-scores > |2| and *p*-values of overlap < 0.05 (and corrected for multiple testing with the Benjamini-Hochberg procedure *p*-values < 0.001).

Core Analysis of DEGs significantly driven by consumption of 70% bean dose indicated involvement of the immune system, whose canonical pathways and functions scored with the highest *z*-scores across the tissues and sex cohorts (Figure 5; Supplementary Tables S2 and S3). Since only 154 analysis-ready DEGs remained in the female subcutaneous fat dataset, this group had the minimum amount of significant functions annotated. In contrast, the male subcutaneous fat group showed a pattern of opposite effects on the biological functions and canonical pathways compared with the mesenteric fat tissue DEGs in both sex cohorts. The most prominent canonical pathways that bean-induced DEGs mapped to are involved in the cellular immune response, cytokine signaling, and pathogen-influenced signaling (Figure 5a). These include Th1/Th2 pathways, leukocyte extravasation signaling, natural killer cell signaling, crosstalk between dendritic cells and natural killer cells, neutrophil extracellular trap signaling pathway, neuroinflammation signaling pathway, T-cell receptor signaling, etc., which showed an overall pattern of being upregulated in the mesenteric fat tissue but significantly downregulated the subcutaneous depot of males. Additionally, bean consumption in the latter group suppressed genes that conventionally participate in phagosome formation, F_cγ_ receptor-mediated phagocytosis in macrophages and monocytes, acute phase response signaling, pathogen-induced cytokine storm signaling, production of nitric oxide and reactive oxygen species in macrophages, and bacteria and viruses pattern recognition pathways. Furthermore, common bean affected DEGs taking part in cancer-associated pathways, such as the SPRINK1 pathway and PD-1/PD-L1 cancer immunotherapy pathway suppression (mesenteric fat), and inhibited S100 family signaling pathway and FAK signaling with upregulated PTEN signaling (subcutaneous fat). The analysis of the most significant biological functions that overlap with bean-stimulated gene expression changes demonstrated a similar pattern of enhancing elements of the immune system in the mesenteric fat depots while reducing thereof in the subcutaneous fat depots (Figure 5b). 

As detailed in Section 2.5, Graphical Summaries were constructed according to heuristic IPA algorithms with 0% bean as the referent group and 70% bean as the experimental group for mesenteric and subcutaneous fat of female and male mice. The top most significant entities from the results of Core Analysis engines are selected into a network: from the Canonical Pathways (by *p*-value ≤ 0.05 of overlap), from Upstream Analysis (by the magnitude of observed differential expression of DEGs (by log_2_ ratio) that matched significant genes, mRNAs, and proteins only with significant *p*-values (≤0.05) and *z*-scores (≥|2|), and from Diseases & Functions with *p*-values ≤ 0.05 and *z*-scores ≥ |2|. Graphical Summary includes inferred relationships (such as Inferred Causation (IC) in presented figures herein by dotted lines) built using IPA’s machine-learning models based on the normalized and weighted values from curated findings from the QIAGEN Ingenuity Knowledge Base.

**Figure 6:**
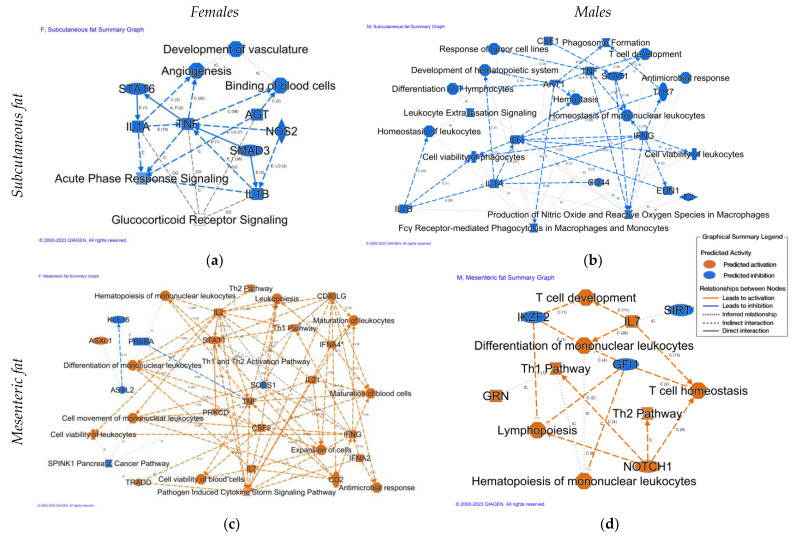
Graphical Summary of dietary bean effects based on DEGs in subcutaneous (**a**,**b**) and mesenteric (**c**,**d**) fat pads of female (**a**,**c**) and male (**b**,**d**) mice. Colors indicate predicted activation (orange) or predicted inhibition (blue). Graphical Summaries were constructed in QIAGEN IPA so as to contain the minimum number of nodes indicating the most significant results.

Figure 6 identifies two observations of particular note: (1) bean consumption upregulates pathways, genes, and functions related to immune function in mesenteric fat of both female and male mice, (2) essentially, all noted pathways, genes, and functions observed in subcutaneous fat are downregulated irrespective of biological sex, and most of these relations either directly or indirectly relate to immune function and its regulation.

## 4. Discussion

The literature on obesity and weight loss is expansive in comparison to that on obesity prevention viewed through the lens of avoiding adult weight gain. While the weight gain avoidance literature primarily focuses on energy balance and the role of physical activity in ameliorating the effects of energy intake above maintenance levels [22], the possibility that a category of food would exert anti-obesogenic activity under conditions of isocaloric intake must be viewed with caution. It is with a recognition of this need for caution that we relate the data presented herein to the scientific literature.

### 4.1. Clinical Relevance and Physiological Considerations

The focus of Section 3.1 is on anthropometric determinants commonly studied in the clinic in the energy balance setting. Figure 1 and Figure 2 graphically demonstrate that body weight gain was dose-dependently reduced in female mice fed *ad libitum* with increasing bean consumption, whereas, in male mice, weight gain was only slowed at the highest level of bean consumption. Body weight data are easily obtained in clinical or population-based studies, and it is the outcome variable for assessing the universal guidance to avoid unintentional adult weight gain. While common speculation in studies of humans has been that pulse consumption promotes satiety [12], reduced caloric intake and body weight gain are not obligatory for the common bean to exert its anti-obesogenic activity, as reported in [14,15]. Rather, a bean-specific effect was observed on fat mass reduction in mice and rats that had similar body weights. To extend our previous observations and ask whether they were applicable when mice were fed *ad libitum*, Table 1 reports final body weight adjusted to linear growth using the tibia length—a measure that is used like BMI in humans [23,24,25]. We also assessed the total mass of body fat as a percent of body weight, which indicated overall effects in the reduction of BMI, and identified the level of bean consumption, at which there is a marked reduction in the assessed fat mass relative to body weight—equivalent to 70% of the total dietary protein. These observations are consistent with the hypothesis that incorporating common bean in the diet both overall suppresses the rate of tissue growth and, at a sufficient dose, specifically reduces lipid accumulation beyond that, which was predicted by the body weight. Importantly, at no point in the study was negative energy balance detected, i.e., no weight loss was observed. Thus, there is no basis for the assertion that common bean will induce loss of either body weight or fatness in obese or overweight individuals. This question is distinct from anti-obesogenic activity considerations discussed herein and needs to be addressed in obese humans and in the obese preclinical models with experimental designs that are standard of practice in clinical studies of weight loss. 

### 4.2. One Size Does Not Fit All

Rather than assess overall fat mass, the data shown in Table 1 and Table 2 and Figure 2 and Figure 3 reflect fat pad-specific effects of bean consumption. Fat depot selection was based on the insightful work reported in [25]. From an energy balance perspective, subcutaneous fat is metabolically adapted to support thermal homeostasis, whereas visceral fat depots are highly plastic and are involved in energy/substrate homeostasis during the fasted versus fed states. As shown in these figures, accumulation of fat in the subcutaneous (inguinal depot) compartment was more responsive to bean consumption than visceral depots considered collectively. This observation suggests the value of further interrogation of subcutaneous fat depots for energy dissipation mechanisms. Scrutiny of the visceral depot data indicates that the mesenteric fat was the most responsive to bean consumption and that the retroperitoneal fat was less responsive in females yet refractory to change in males. While the meaning of these findings and their metabolic relevance is unclear, the impact on the mesenteric depot provides for further interrogation of how bean-mediated effects on the gut microbial community [26,27] and, possibly, the immune system may be linked to effects recently reported on the lipid metabolism in the liver [16]. 

Another insight was revealed when we focused on the distribution of data points in the box plots for subcutaneous and mesenteric fats across the bean dose groups (Figure 2 and Figure 3). Evidence for responder and non-responder subgroups of animals within each treatment group was supported statistically. This included both the bean-free control and the bean-containing groups. First, the existence of subgroups in the control is consistent with the widely recognized variation in the dietary induced obesogenic phenotype in C57BL6/J mice [28,29,30]. The distributions in the bean-fed groups suggest that different amounts of bean in the diet may exert beneficial effects depending on responsivity and that this extends across biological sex despite the fact that female mice are more responsive, overall, in experiencing anti-obesogenic activity. These findings also indicate that divergent bi-directional breeding for bean sensitivity could be used to unmask genetic determinants of responsiveness. This genetic selection approach has been done for both body weight gain and for inherent aerobic capacity in preclinical models [31,32]. Such experiments could provide genetic determinants that can be used to formulate precision strategies for weight gain avoidance. This is a novel example of nutrigenomics.

### 4.3. Underlying Mechanisms

The value of a hybrid approach for identifying candidate mechanisms is that it provides information for the generation of alterative hypotheses. The hypothesis-driven component of our analysis focused on genes and proteins involved in the energy regulation and lipid metabolism, which have been implicated in the development of obesity [33,34]. However, at the level of protein expression, no significant effects of bean consumption on PPARγ, FASN, or SCD were observed (Table 3). This was contrary to our initial expectations; however, given that the effects of bean consumption on weight gain were gradual over time, the lack of marked effects on regulators and enzymes involved in lipid metabolism measured as a snapshot in time is not surprising. Because of these negative findings, this line of investigation was not further pursued. The goal of the data-driven component of our investigation was to use the RNA-Seq analysis to provide an overview of the functional endpoints implicated in the dietary bean-induced effects. Comparative analyses of bean-driven DEGs permitted us to detect similarities and differences in common bean effects across the adipose depots and sex cohorts of mice. 

Only three DEGs were commonly differentially expressed upon bean consumption in both fat pads of both sex cohorts. Serum amyloid A, encoded by *Saa3*, is a marker of recruitment, adhesion, and accumulation of monocytes and macrophages associated with the hypertrophic adipocytes, secreting it in the setting of obesity and the corresponding local and systemic inflammation [35]. However, there is also evidence that amyloid A does not play a causative role in the development of obesity [36]. Consumption of bean significantly suppressed the expression of *Saa3* in all experimental groups. Obesity-associated increased production of serum amyloid A is accompanied by the overproduction of hyaluronan that, together with monocyte chemoattractant CCL2, exhibit similar functions [35]. Female mice fed bean showed the reduced expression of mesenteric hyaluronidase-1 (*Hyal1*), an enzyme that degrades hyaluronan in the lysosomes. In contrast, males showed upregulated mesenteric *Lyve1*—a hyaluronan receptor 1 on the subpopulation of macrophages in the lymphatic vasculature associated with adipose tissue where they are involved in the lipid storage control [37]. Additionally, bean-fed female and male mice showed the reduced expression of *Ccl2* in the mesenteric and subcutaneous fat pads, respectively. Such a pattern is consistent with the observed evidence of active participation of both humoral and cellular components of the immune system in mediating the anti-obesogenic effects of beans. 

Another common DEG affected by bean consumption in all groups was *Timp4,* exhibiting sex-dependent effects as it was upregulated in males but downregulated in females regardless of fat pad localization. TIMP4 is one of the endogenous inhibitors of metalloproteinases associated with remodeling of the extracellular matrix and expandability of the adipose tissue during the development of obesity. TIMP4, in particular, was previously shown to be reduced in the visceral fat of obese mice, even though its deletion protects mice from diet-induced obesity [38,39]. It is also involved in the regulation of insulin sensitivity. A similar discrepancy in *Timp4* levels based on biological sex has been reported: mRNA levels were lower in females than in males under low-fat diet, but the high-fat diet challenge nullified this difference for both subcutaneous and mesenteric depots by silmutaneously decreasing its gene expression in males (also detected in epididymal fat) but increasing it in females [40]. Another study also stated that a high-fat diet suppresses the transcription of *Timp4* in the perigonadal fat pad of males [41]. In turn, protein levels of TIMP4 in subcutaneous fat were markedly higher in females than in males, but again a high-fat diet removed such a sex-dependent difference, demonstrating protein reduction in females without any changes in males [40]. Interestingly, the authors also reported sex differences for another metalloproteinase inhibitor, *Timp1,* whose mRNA concentration was increased in all adipose depots in males upon high-fat diet feeding but not in females. This abolished the discrepancy with females in subcutaneous fat, whose *Timp1* mRNA levels were initially higher under low- but comparable under high-fat diet. In mesenteric and perigonadal fats comparison, *Timp1* mRNA levels did not differ between females and males but high-fat feeding resulted in 4 times higher levels thereof in the latter cohort [40]. This inhibitor of matrix metalloproteinases was shown to be elevated in the circulation of patients with metabolic diseases, including metabolic-associated fatty liver disease, diabetes, and obesity [39]. This finding is consistent with our observation of anti-obesogenic effects of bean consumption, which statistically reduced *Timp1* in the mesenteric fat of both female and male mice. Adipose tissue-associated macrophages produce elastase MMP12—one of the targets of TIMP1 and coincidentally a marker of inflammatory response during obesity. A high-fat diet reportedly promoted its expression in all the fat pads of male but not female mice, enhancing even greater sex-dependent difference between them [40]. Correspondingly, in our study, common bean significantly reduced *Mmp12* RNA levels in both mesenteric and subcutaneous fat depots of male mice only. 

Finally, *Ubd* encoding for ubiquitin D is induced by interferon γ (IFNγ) and tumor necrosis factor α (TNFα) and via nuclear factor kappa B (NF-κB) signaling. *Ubd* promotes inflammation, and is associated with fatty liver, hepatocellular carcinoma, and crosstalk between obesity and diabetes [42,43]. However, both these reports indicate contradicting evidence on the upregulation or downregulation of *Ubd* levels in the obesity setting. Another report highlights the critical role of ubiquitin D in the lipogenesis of the porcine intramuscular and subcutaneous preadipocytes, as well as overall regulatory function in the cell development, growth, proliferation, and even apoptosis [44]. We report that the consumption of beans suppressed *Ubd* expression in the investigated fat tissues of males, in contrast to females where its levels were reduced in the subcutaneous but enhanced in the mesenteric fat. Therefore, *Ubd* involvement in diet-induced obesity pathogenesis and prevention requires further investigation. 

Nevertheless, these results strengthen the common trend observed with DEGs induced by common bean in the diet. Following the patterns of canonical pathways and biological functions associated with the bean-induced DEGs and further recapitulated by the QIAGEN IPA-algorithmized Graphical Summaries, it was clear that neither the lipid metabolism, nor the energy balance regulatory pathways, were dominating bean-mediated effects in either fat pad type for either female or male mice. This finding is consistent with the negative protein expression data reported in Table 3. Instead, multiple components involved in immune system function were differentially induced in the mesenteric fat pad of female and male mice with common bean consumption. In contrast, the downregulation thereof was observed in the subcutaneous fat depot, particularly in male mice. Obesity-related alterations in the immune system have been reported for both the visceral and subcutaneous fat depots [45,46,47]. Moreover, the induction of immune function in the mesenteric fat pad is consistent with literature expectations [48], particularly in view of our recent report that 70% of the total dietary protein from the common bean relandscapes the composition of the intestinal microbiota [26]. However, in-depth analysis of the hypotheses related to immune function will require new animal experiments with different approaches, e.g., flow cytometry. As such, those investigations are beyond our current work scope; however, the findings summarized in Figure 6 will drive that initiative. 

### 4.4. Strengths and Limitations

The strengths of this study include the dose response study design, the investigation of both male and female mice, the large number of mice per group that provided insights about common bean responder and non-responder populations, the evaluation of both subcutaneous and multiple visceral adipose depots, and the large number of tissue samples subjected to the RNA-Seq analysis such that both overall effects and differences within a treatment group defined as subpopulations could be investigated. Other than the often cited and appropriate concern that mice are not people, which indicates the need for caution in translating our findings to human populations, we chose to focus on gene expression profiles for the identification of candidate tissue markers of response with limited validation using advanced techniques for quantifying protein expression. It is important to recognize that gene expression does not necessarily correlate with protein expression, but this was considered in focusing on genes with higher (>10) counts per million. This strategy also recognizes that detailed proteomic and metabolomic analyses are required to identify candidate mediators of anti-obesogenic activity and their target gene products. The work reported here provides a focus for those multi-omics analyses that are considered beyond the scope of the work reported herein.

## 5. Conclusions

Despite the limitations that have existed in quantifying pulse intake in human populations, consumption is low in most of the developed countries and intakes generally decrease in developing countries as socioeconomic status improves. Low pulse consumption, which is a reversal of the historical role of this crop type that began before the agricultural revolution, is an unappreciated companion of the obesity pandemic. The work reported herein suggests a path forward for critical tests of the anti-obesogenic activity of pulses in human populations. Specifically, such studies must recognize that *one size does not fit all*. Experimental designs must consider that the pulse dose in the diet is a critical consideration and will likely vary between biological sexes, and that within a cohort, subpopulations of responders and non-responders are likely to exist. Before clinical/population studies proceed, the opportunity for critical insights will be enhanced if the chemicals that pulse consumption induces in the host and the targets of those chemicals that account for anti-obesogenic activity are identified. The molecular work reported herein provides potential candidate targets and sets the stage for metabolomic analyses to identify chemical mediators. With that knowledge in hand, robustly designed clinical and population studies can be undertaken with true potential to unearth food-based precision approaches to avoid adult weight gain that will improve human well-being and reduce chronic disease risk.

## Figures and Tables

**Table 1:** Anthropometric data at the end of the study.

*Females*					
Percent of the Total Dietary Protein from Beans	Body Weight, g	Body Mass Index, g/mm	Percent Body Fat, %	Subcutaneous Fat Mass, g/mm	Visceral Fat Mass, g/mm
0	36.3 ± 0.6 ^a^	2.1 ± 0.03 ^a^	3.8 ± 0.1 ^a^	106 ± 4 ^a^	205 ± 7 ^a^
17.5	36.4 ± 1.0 ^a^	2.1 ± 0.05 ^a^	4.0 ± 0.1 ^a^	106 ± 7 ^a^	208 ± 11 ^a^
35	33.2 ± 0.9 ^b^	1.9 ± 0.05 ^a^	3.6 ± 0.1 ^a^	93 ± 7 ^a^	175 ± 12 ^a^
70	28.8 ± 0.7 ^c^	1.7 ± 0.04 ^b^	2.6 ± 0.1 ^b^	59 ± 5 ^b^	119 ± 10 ^b^
Low-fat Control	25.6 ± 0.6 ^c^	1.5 ± 0.04 ^b^	2.2 ± 0.1 ^c^	46 ± 4 ^b^	76 ± 8 ^b^
***Males***					
**Percent of the Total** **Dietary Protein from Beans**	**Body Weight,** **g**	**Body Mass Index,** **g/mm**	**Percent** Body Fat, **%**	**Subcutaneous Fat Mass,g/mm**	**Visceral Fat Mass,** **g/mm**
0	49.2 ± 0.6 ^a^	2.8 ± 0.05 ^a^	4.1 ± 0.1 ^a^	132 ± 6 ^a^	196 ± 6 ^a^
17.5	50.7 ± 0.6 ^a^	2.9 ± 0.03 ^a^	4.3 ± 0.1 ^a^	131 ± 4 ^a^	198 ± 5 ^a^
35	49.1 ± 0.7 ^a^	2.7 ± 0.04 ^a^	4.2 ± 0.1 ^a^	120 ± 6 ^a^	196 ± 7 ^a^
70	44.3 ± 0.7 ^b^	2.5 ± 0.05 ^b^	3.5 ± 0.1 ^b^	90 ± 7 ^b^	198 ± 7 ^a^
Low-fat Control	42.4 ± 0.7 ^b^	2.4 ± 0.04 ^b^	3.7 ± 0.1 ^b^	93 ± 3 ^b^	188 ± 4 ^a^

Values are Means ± SEM, *n* = 20 female/male mice per group. Different letter superscripts denote statistically different values within a column per sex cohort, *p* < 0.05. Body mass index (BMI) = body weight/tibia length. Percent body fat = (subcutaneous + visceral fat)/body weight; note that it is not the total body fat. Subcutaneous fat mass = inguinal fat depot. Visceral fat mass = perigonadal + mesenteric + retroperitoneal fat depots.

**Table 2:** Visceral fat mass data at the end of the study.

Percent of the Total Dietary Protein from Beans	Mesenteric Fat Mass, mg/mm		Perigonadal Fat Mass, mg/mm		Retroperitoneal Fat Mass, mg/mm	
	Females	Males	Females	Males	Females	Males
0	35.2 ± 3.1 ^a^	65.8 ± 3.1 ^a^	147.0 ± 6.1 ^a^	102.9 ± 6.1 ^a^	22.9 ± 0.9 ^a^	27.9 ± 0.9 ^a^
17.5	39.0 ± 3.1 ^a^	69.5 ± 3.1 ^a^	147.0 ± 6.1 ^a^	100.7 ± 6.1 ^a^	22.0 ± 0.9 ^a^	28.2 ± 0.9 ^a^
35	32.9 ± 3.1 ^a^	61.9 ± 3.1 ^a^	123.4 ± 6.1 ^a^	106.9 ± 6.1 ^a^	18.6 ± 0.9 ^a,b^	27.4 ± 0.9 ^a^
70	23.5 ± 3.1 ^b^	44.6 ± 3.1 ^b^	82.1 ± 6.1 ^b^	124.8 ± 6.1 ^b^	13.2 ± 0.9 ^c^	29.1 ± 0.9 ^a^
Low-fat Control	15.3 ± 3.6 ^b^	41.5 ± 3.1 ^b^	52.1 ± 7.1 ^b^	120.1 ± 6.1 ^b^	9.0 ± 1.1 ^c^	26.1 ± 0.9 ^a^
	Factorial ANOVA					
	Diet, *p* < 0.001 Sex, *p* < 0.001		Diet, *p* < 0.001 Sex, *p* = 0.8		Diet, *p* < 0.001 Sex, *p* < 0.001	

Values are Means ± SEM, *n* = 20 mice per group. Different letter superscripts denote statistically different values within a column, *p* < 0.05.

**Table 3:** Effect of the common bean on the targeted protein expression.

Protein	Fat Mass Tissue	Female, Normalized AU		Male, Normalized AU		*Factorial ANOVA*
		*0%*	*70%*	*0%*	*70%*	
PPARγ	Mesenteric	82.6 ± 26.4	59.0 ± 26.4	153.1 ± 26.4	96.9 ± 26.4	*Diet, p* = *0.08;* *Sex, p* = *0.058;* *Tissue, p < 0.001*
		*p* = 0.12		*p* = 0.08		
	Subcutaneous	211.6 ± 26.4	195.5 ± 26.4	241.9 ± 26.4	205.4 ± 26.4	
		*p* = 0.6		*p* = 0.5		
SCD	Mesenteric	41.9 ± 32.6	69.4 ± 32.6	16.6 ± 2.0 ± 2.0	12.0 ± 2.0	*Diet, p* = *0.65;* *Sex, p* = *0.099;* *Tissue, p = 0.047*
		*p* = 0.6		*p* = 0.13		
	Subcutaneous	9.8 ± 1.1	10.6 ± 1.1	14.1 ± 2.0	10.3 ± 2.0	
		*p* = 0.62		*p* = 0.19		
FASN	Mesenteric	31.5 ± 11.3	37.3 ± 11.3	14.4 ± 5.7	7.8 ± 5.7	*Diet, p* = *0.51*; *Sex, p* = *0.20*; *Tissue, p < 0.001*
		*p* = 0.7		*p* = 0.4		
	Subcutaneous	1900 ± 337	1676 ± 337	1489 ± 313	1282 ± 313	
		*p* = 0.7		*p* = 0.6		

Values are standardized means ± SEM derived from the factorial ANOVA model.

## Data Availability

The RNA sequencing data reported herein is being submitted to the Gene Expression Omnibus, a database for gene expression profiling managed by the National Center for Biotechnology Information. Please contact the corresponding author for the GEO accession number.

## Supplementary Materials

The following supporting information can be downloaded at: https://www.mdpi.com/article/10.3390/nu15092015/s1, Table S1: Diet formulations; Table S2: Canonical pathways; Table S3: Diseases & Functions. Figure S1: Histological sections of the subcutaneous and mesenteric fat depots; Figure S2: Box plots for expression of genes involved in lipid metabolism; Figure S3: Western blot electropherograms for proteins involved in lipid metabolism.
