# Supplementary material for: Consumption of Common Bean Suppresses the Obesogenic Increase in Adipose Depot Mass: Impact of Dose and Biological Sex
Source: Nutrients. 2023 Apr 22;15(9):2015. doi: 10.3390/nu15092015 (PMC10180429; doi:10.3390/nu15092015)
Supplement: Supplementary file 1 [file nutrients-15-02015-s001.zip › Supplementary Figures S1-S3.pdf]

Supplementary materials

Figure S.1.a. Histological sections of subcutaneous fat depot. Bar is 5 microns: a and b: female mice; c and d: male mice. Panels a and c: 0% bean; panels b and d: 70% protein from bean.

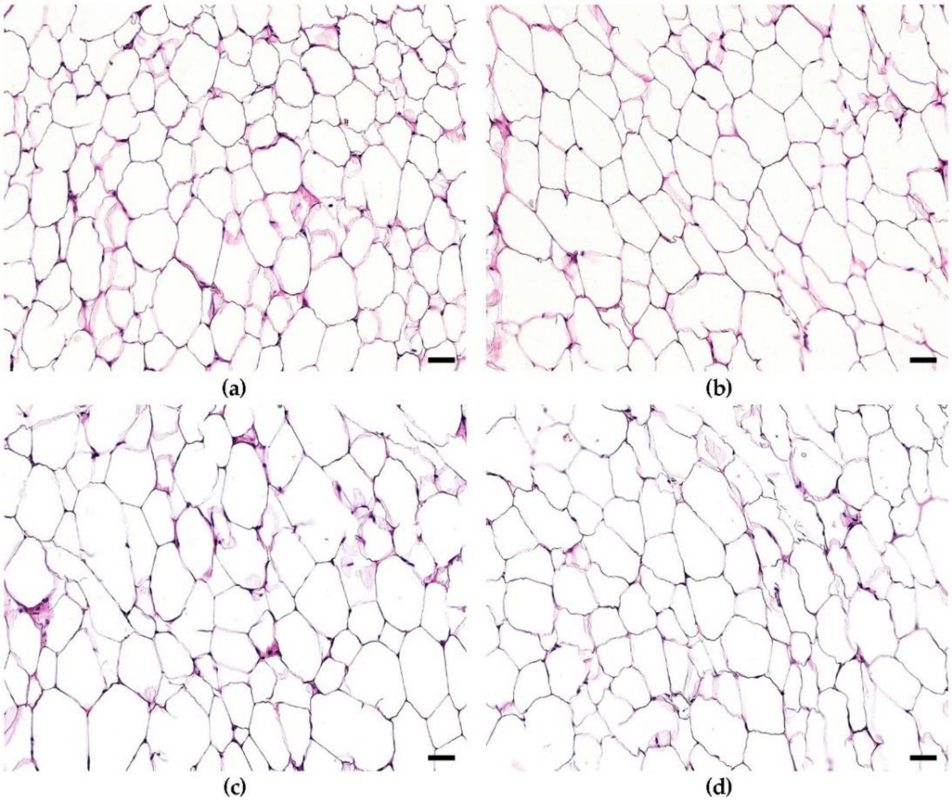

Figure S1.b. Histological section of mesenteric fat depot.

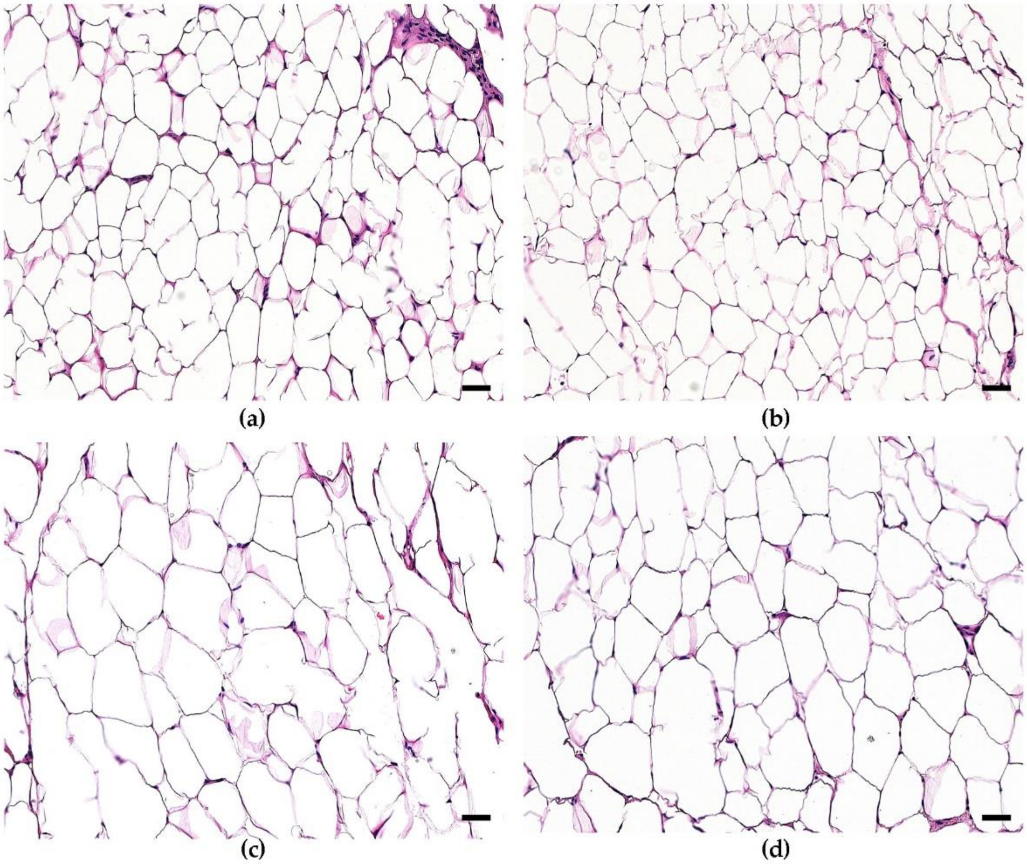

Figure S2. Box plots for expression of genes involved in lipid metabolism.

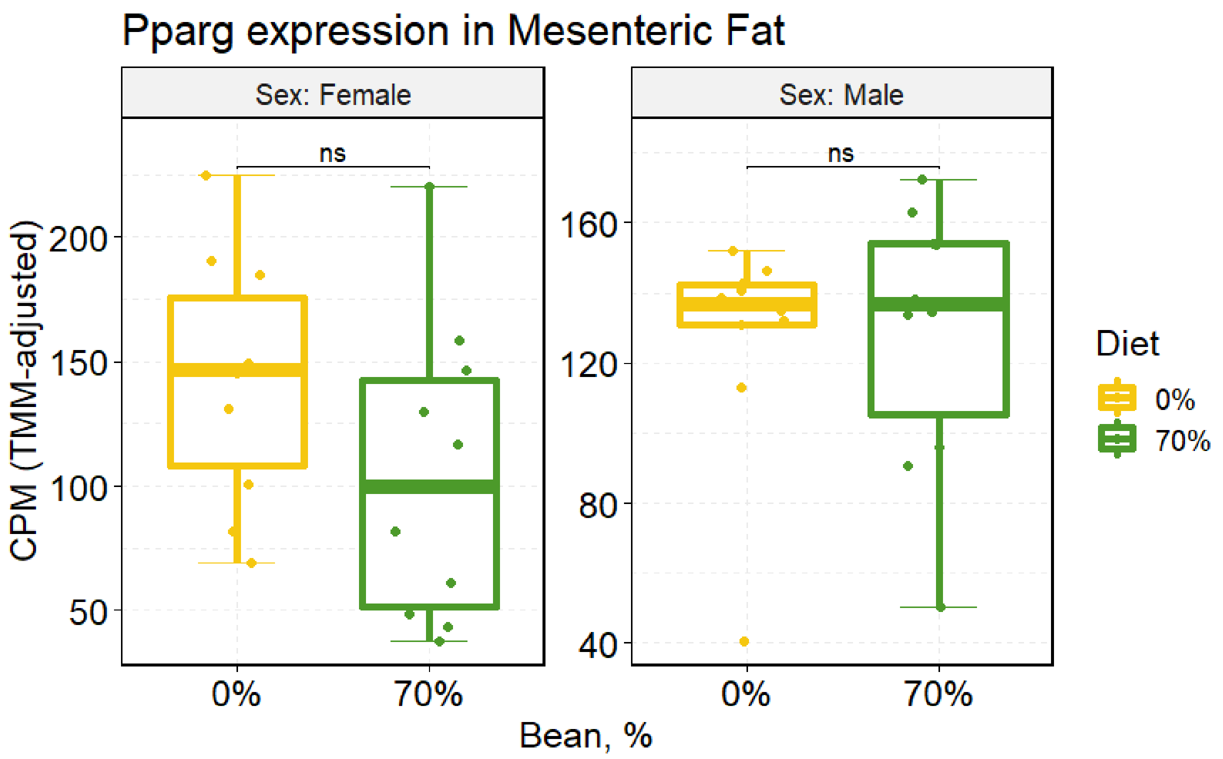

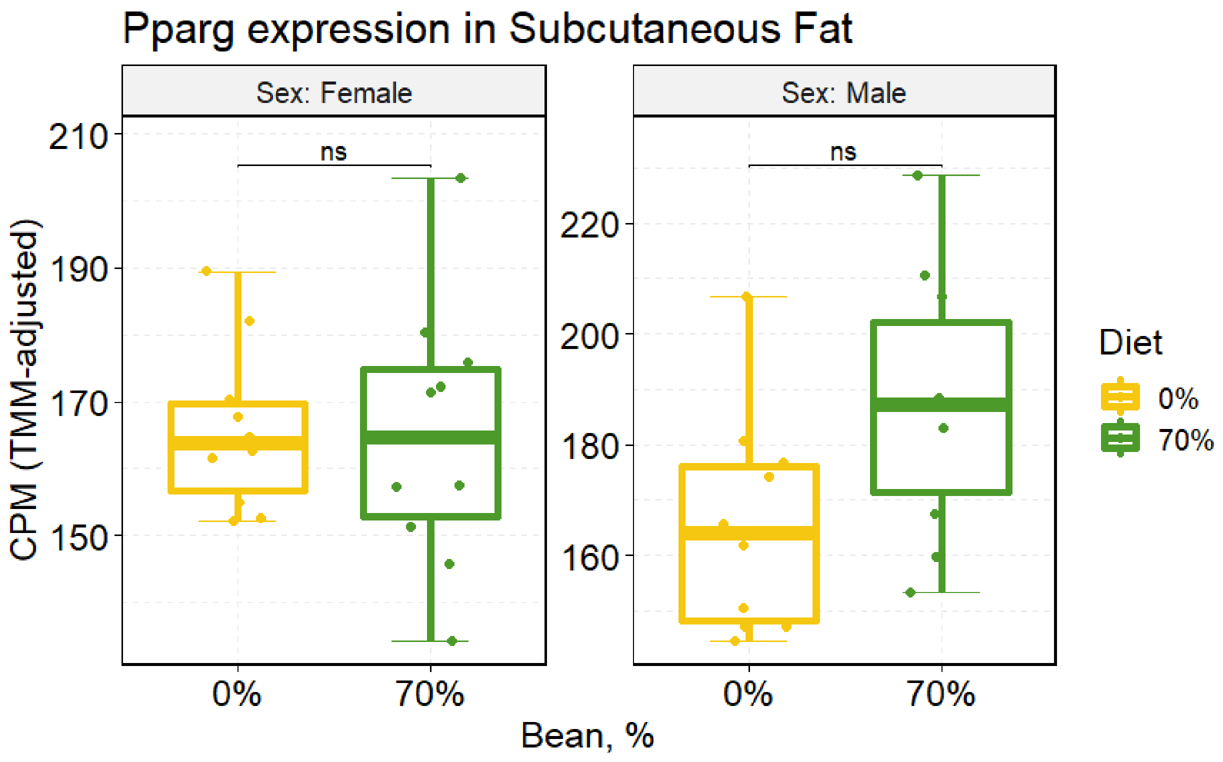

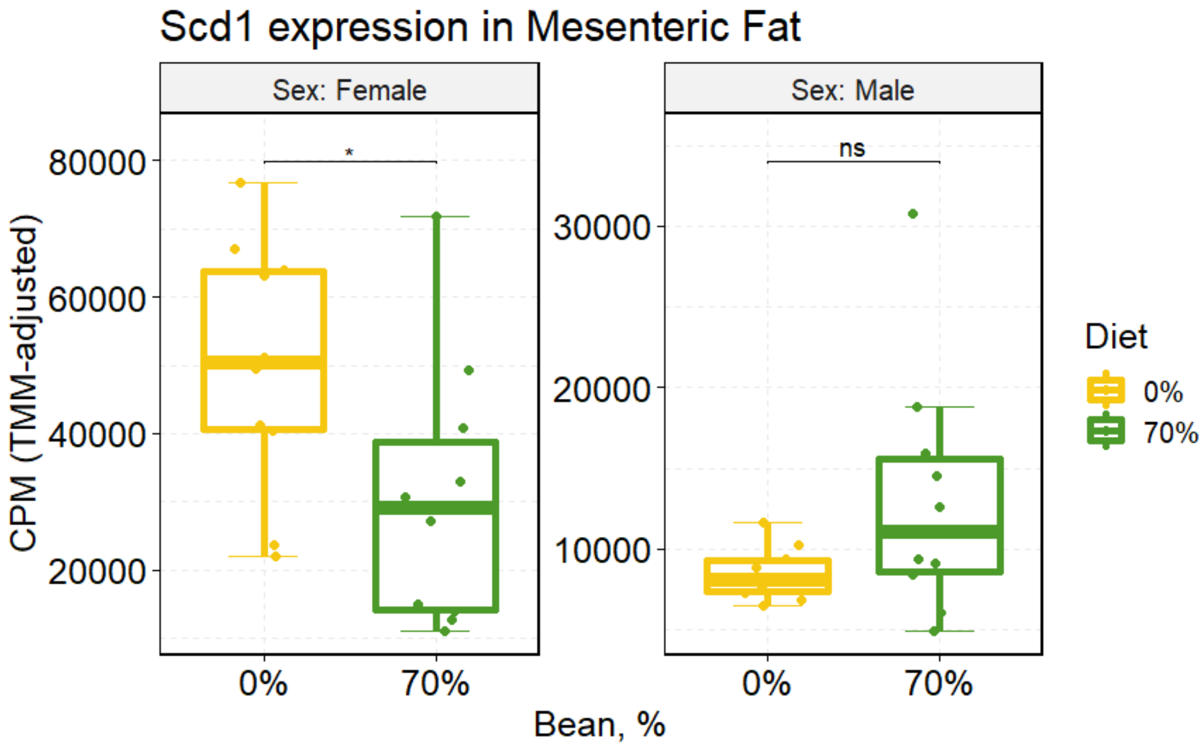

Scd1 expression in Mesenteric Fat
Sex: Female
Sex: Male
*
ns
80000
60000
40000
20000
30000
20000
10000
CPM (TMM-adjusted)
0%
70%
0%
70%
Bean, %
Diet
0%
70%

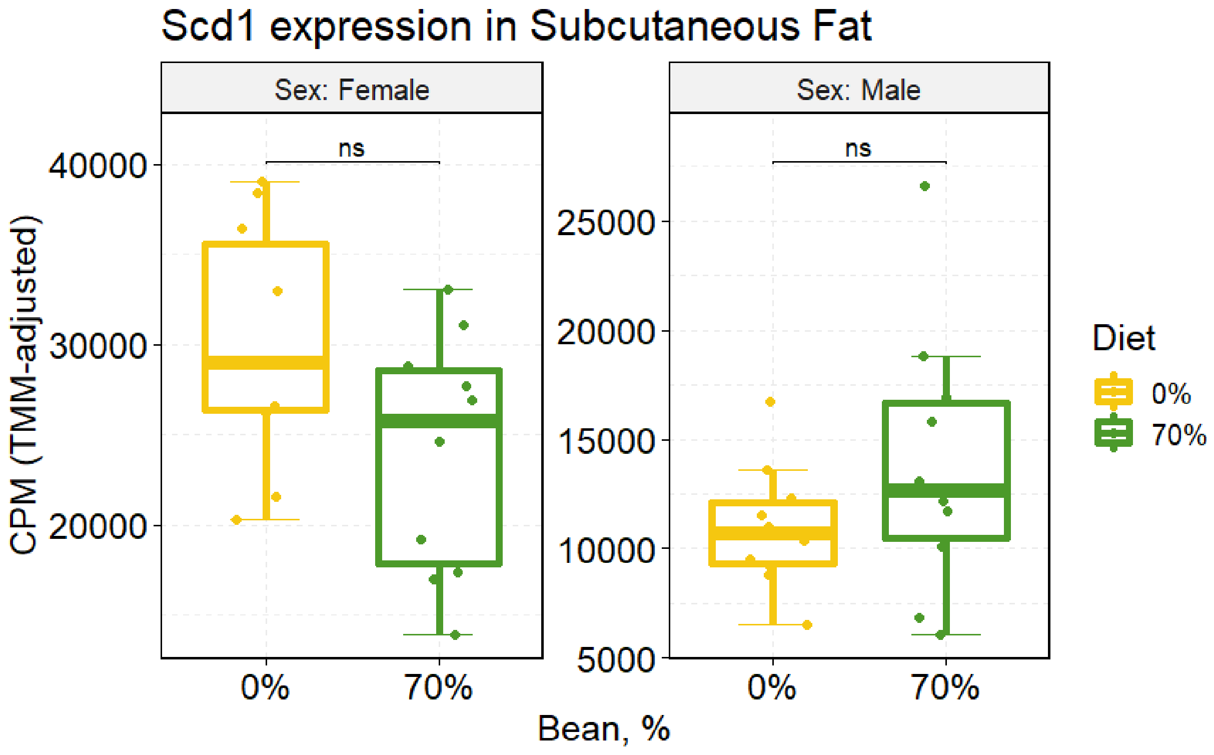

Scd1 expression in Subcutaneous Fat
Sex: Female
Sex: Male
ns
ns
40000
30000
20000
25000
20000
15000
10000
5000
CPM (TMM-adjusted)
0%
70%
0%
70%
Bean, %
Diet
0%
70%

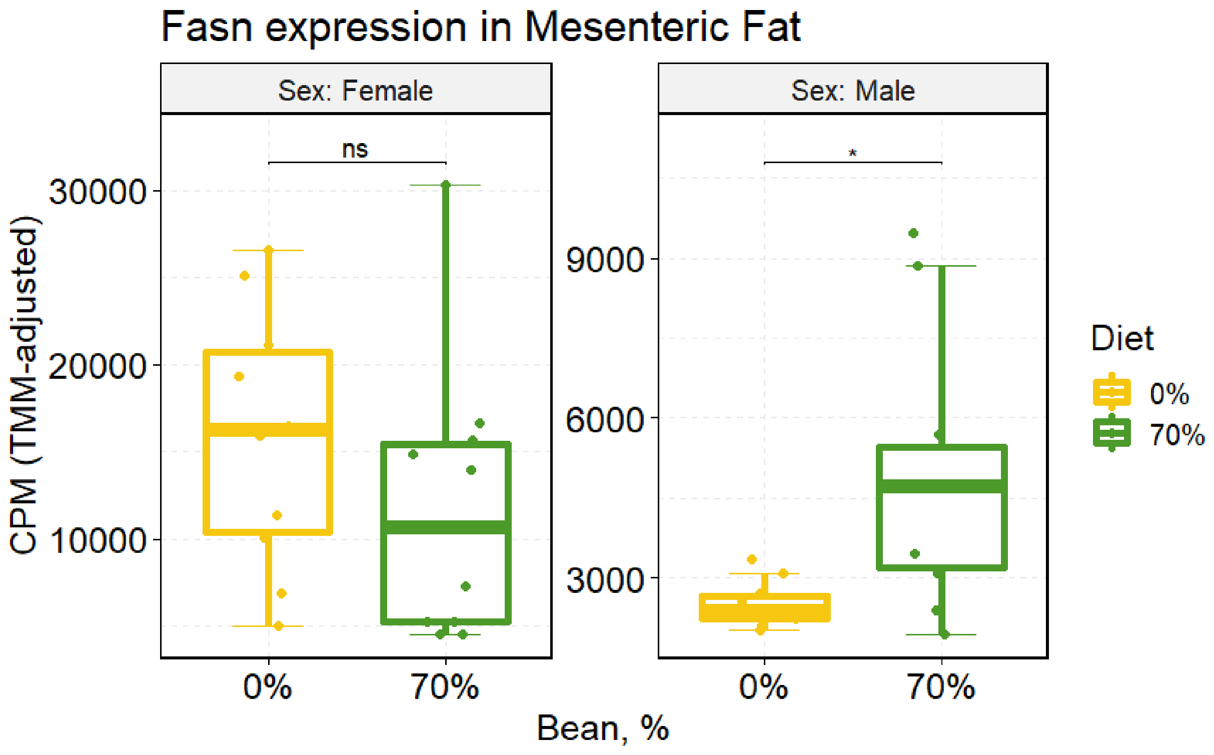
Fasn expression in Mesenteric Fat
Sex: Female
Sex: Male
ns
*
CPM (TMM-adjusted)
30000
20000
10000
9000
6000
3000
0%
70%
Bean, %
Diet
0%
70%

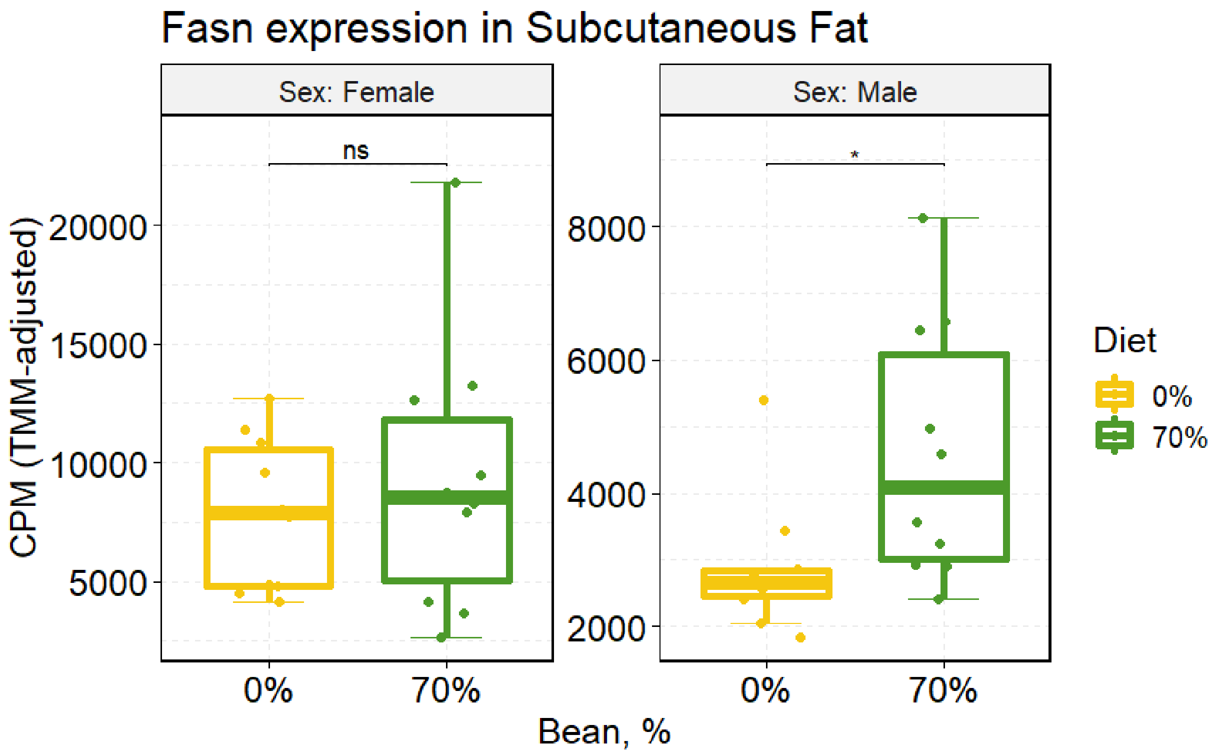
Fasn expression in Subcutaneous Fat
Sex: Female
Sex: Male
ns
*
CPM (TMM-adjusted)
20000
15000
10000
5000
8000
6000
4000
2000
0%
70%
Bean, %
Diet
0%
70%

Figure S3

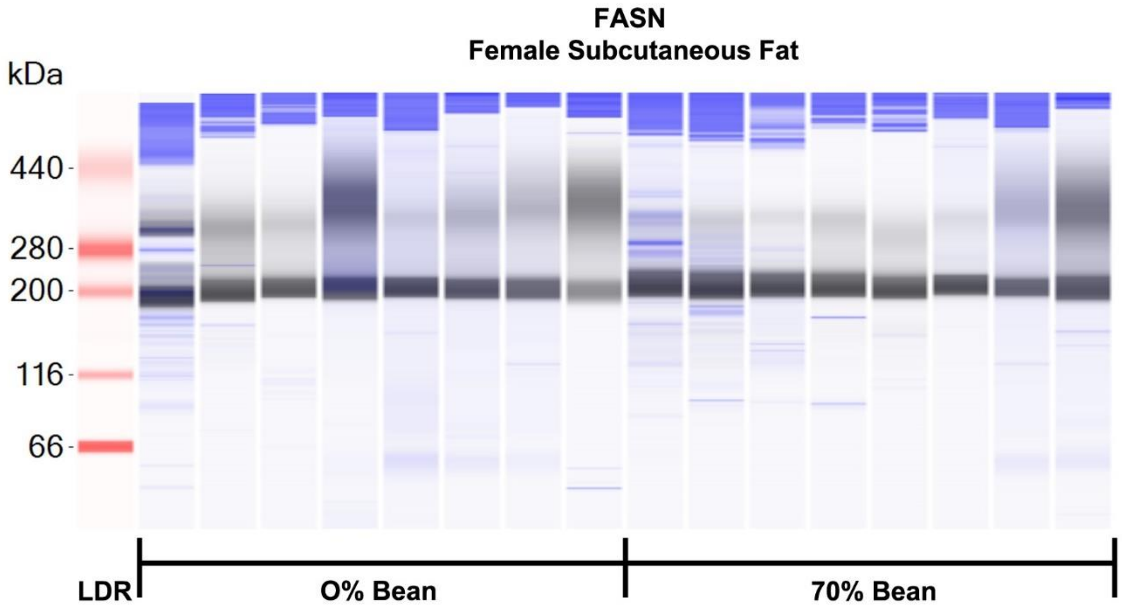
FASN
Female Subcutaneous Fat
kDa
440
280
200
116
66
LDR
O% Bean
70% Bean

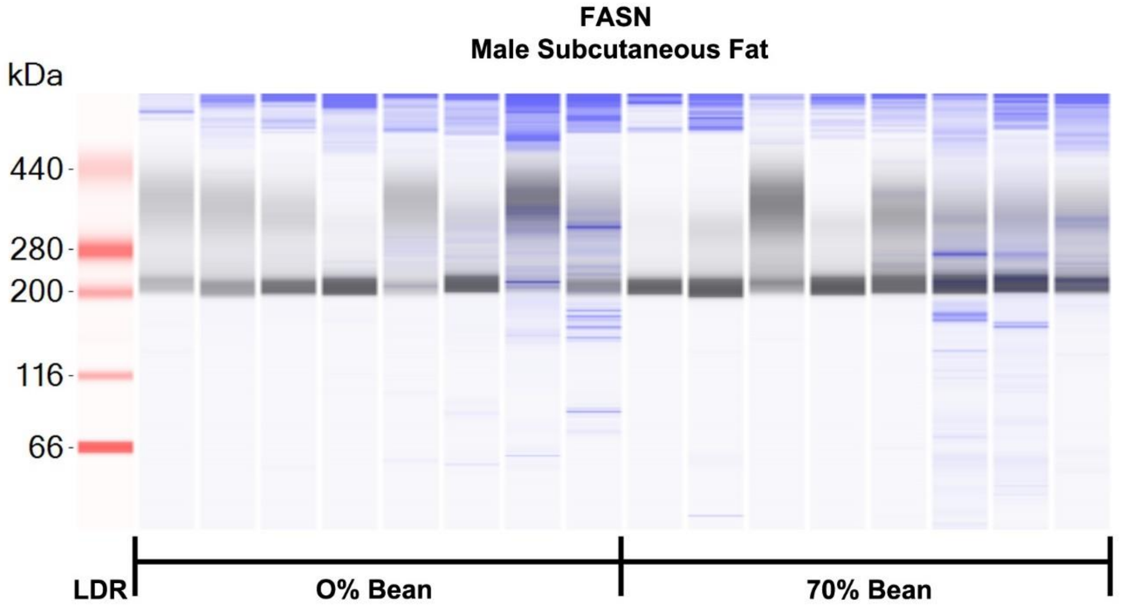
FASN
Male Subcutaneous Fat
kDa
440
280
200
116
66
LDR
O% Bean
70% Bean

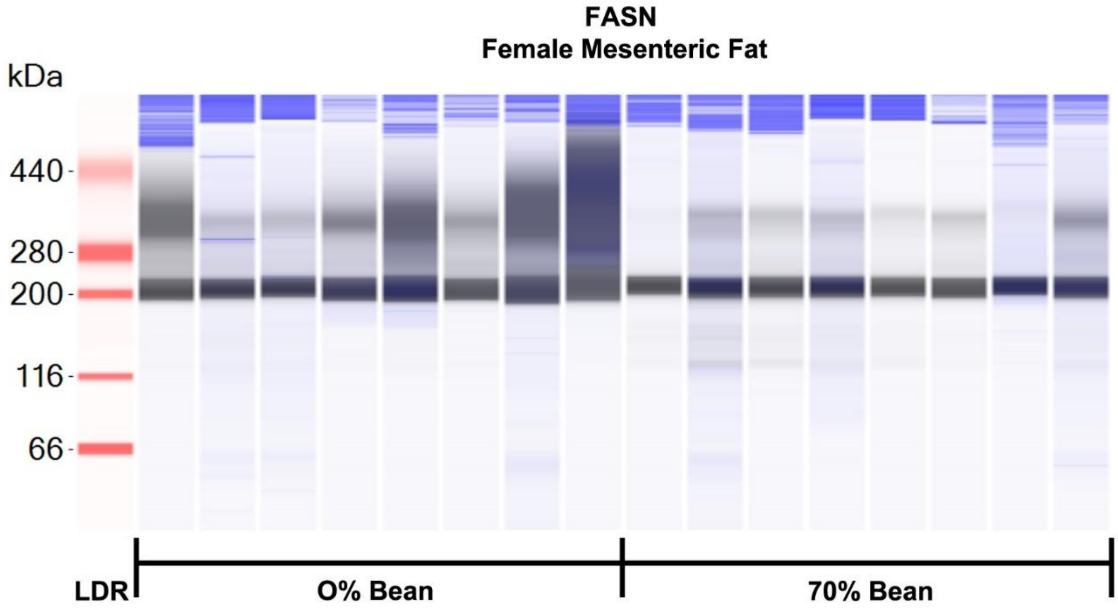
FASN
Female Mesenteric Fat
kDa
440
280
200
116
66
LDR
O% Bean
70% Bean

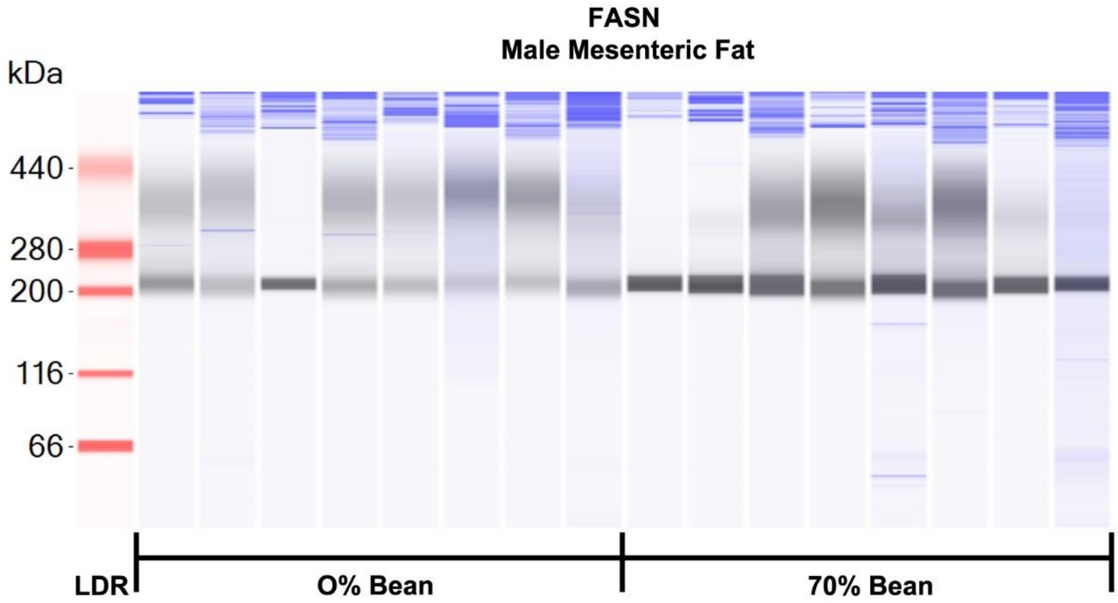
FASN
Male Mesenteric Fat
kDa
440
280
200
116
66
LDR
O% Bean
70% Bean

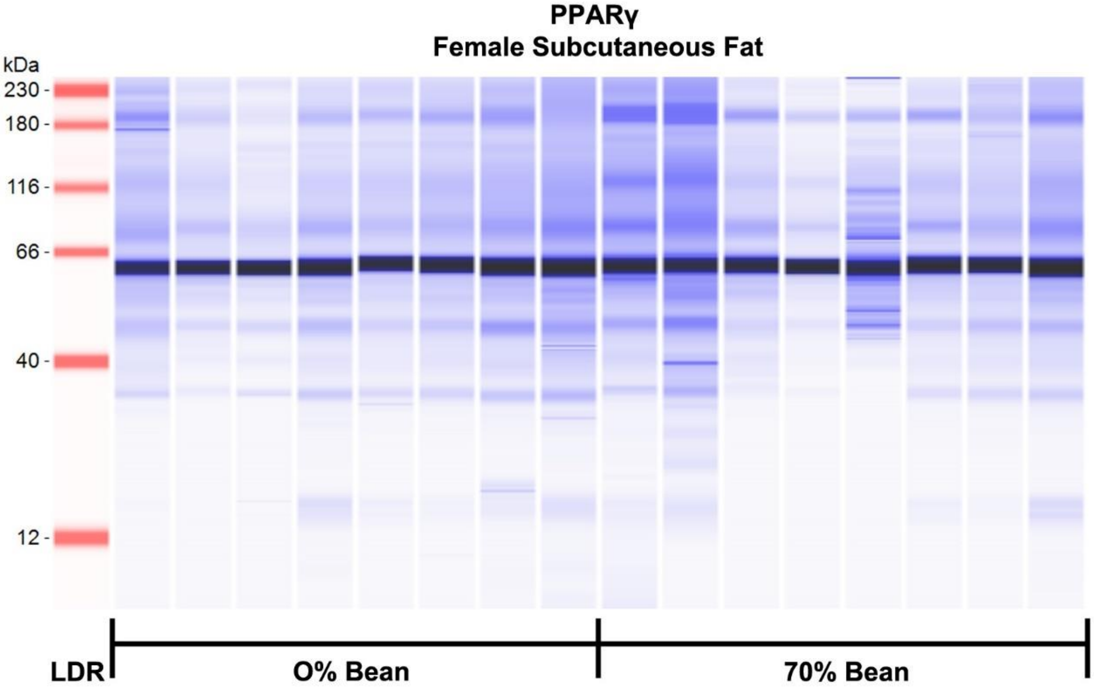
PPARγ
Female Subcutaneous Fat
kDa
230
180
116
66
40
12
LDR
O% Bean
70% Bean

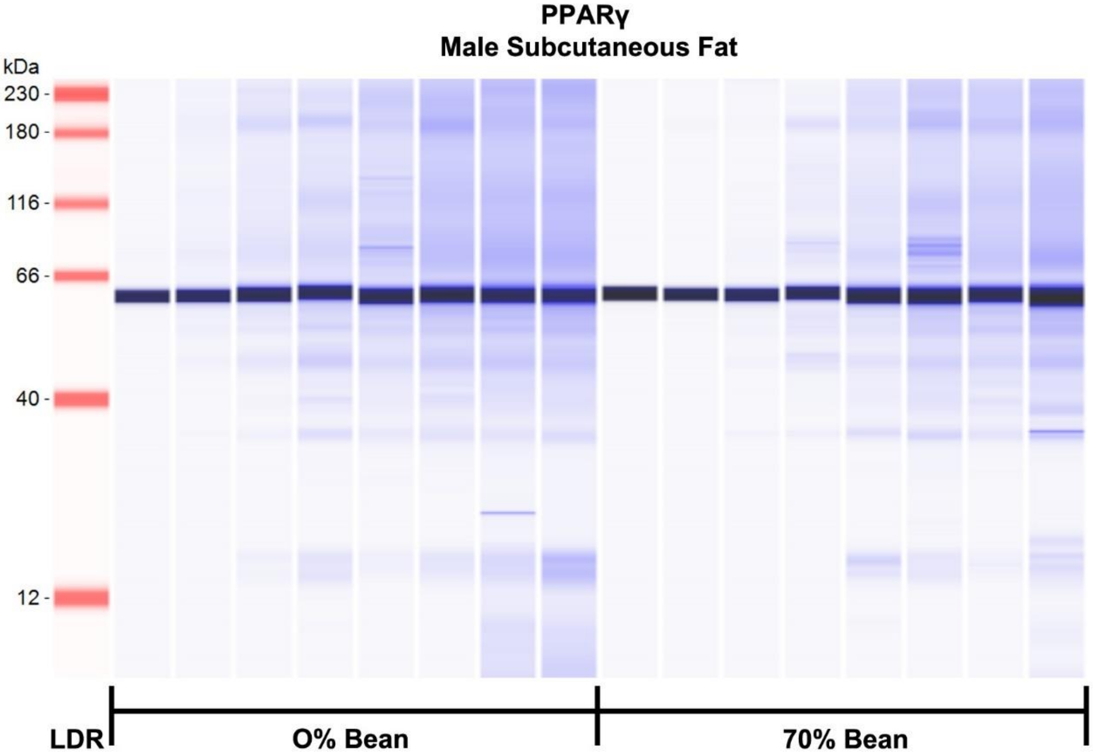
PPARγ
Male Subcutaneous Fat
kDa
230
180
116
66
40
12
LDR
O% Bean
70% Bean

PPARγ
Female Mesenteric Fat

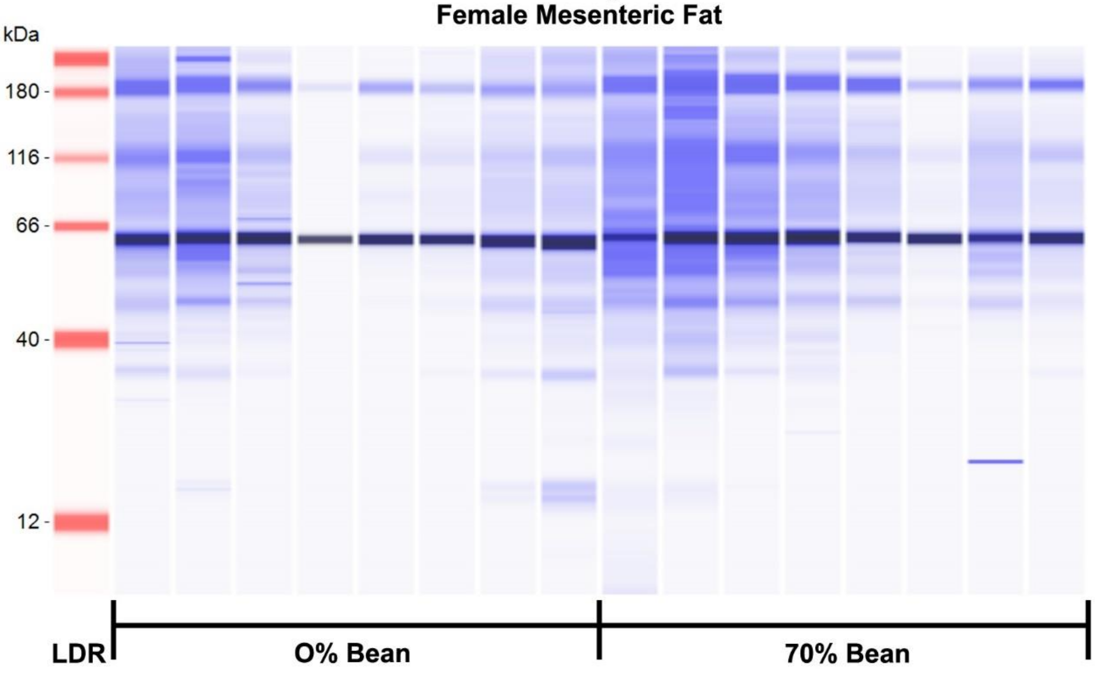

PPARγ
Male Mesenteric Fat

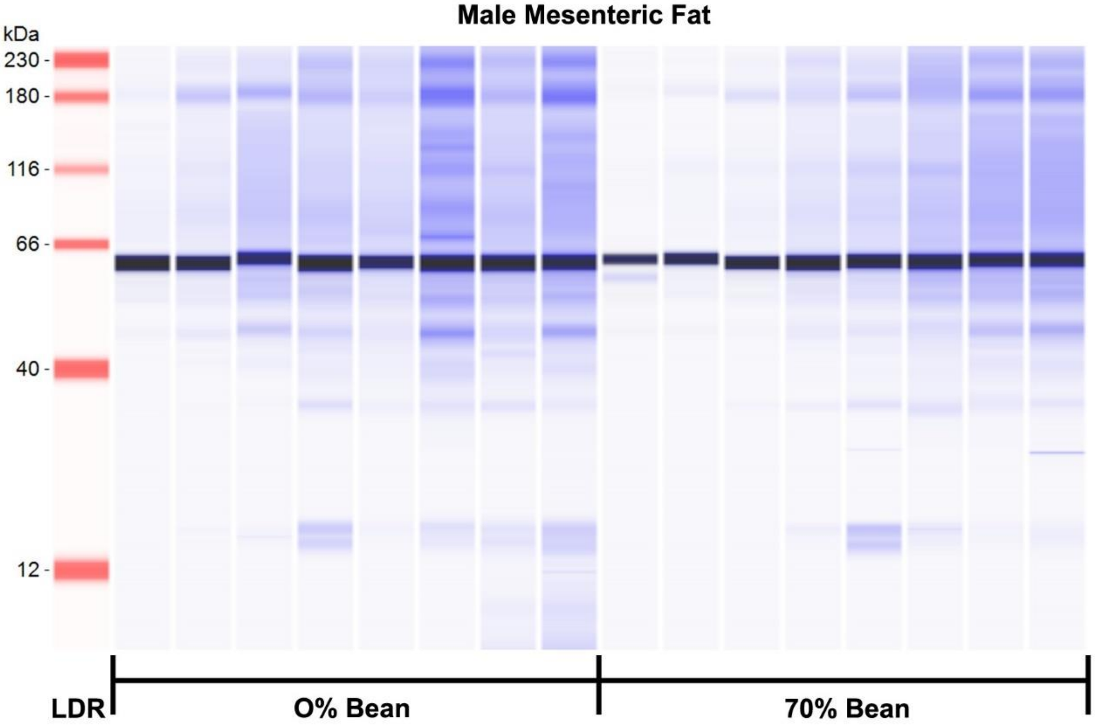

SCD
Female Subcutaneous Fat

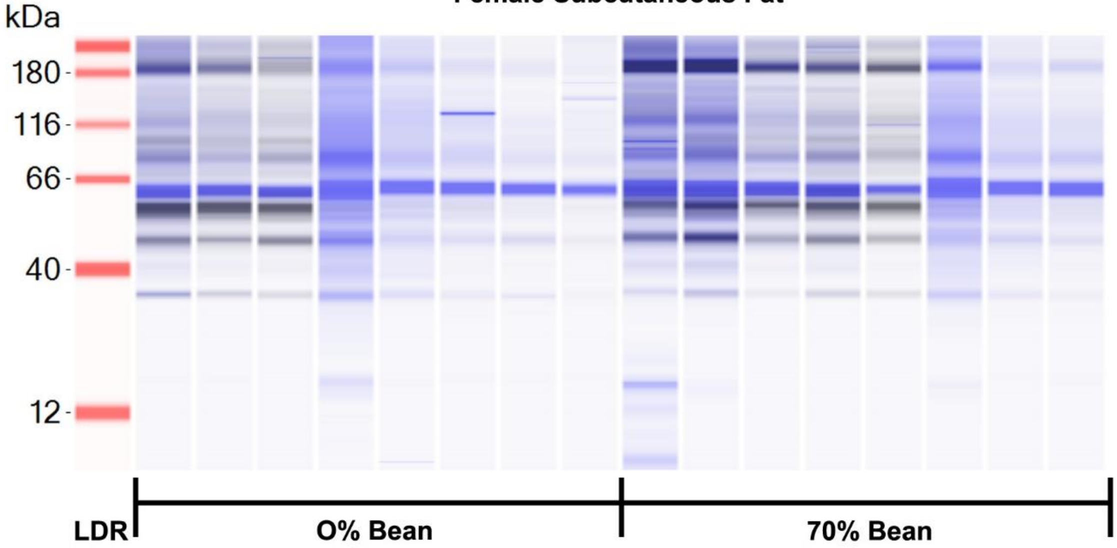

SCD
Male Subcutaneous Fat

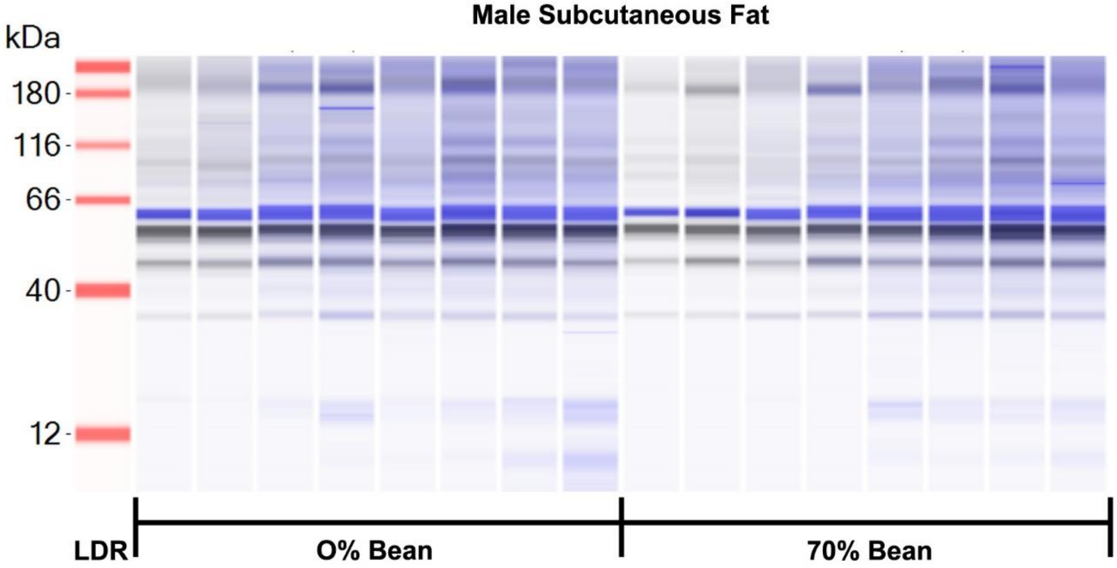

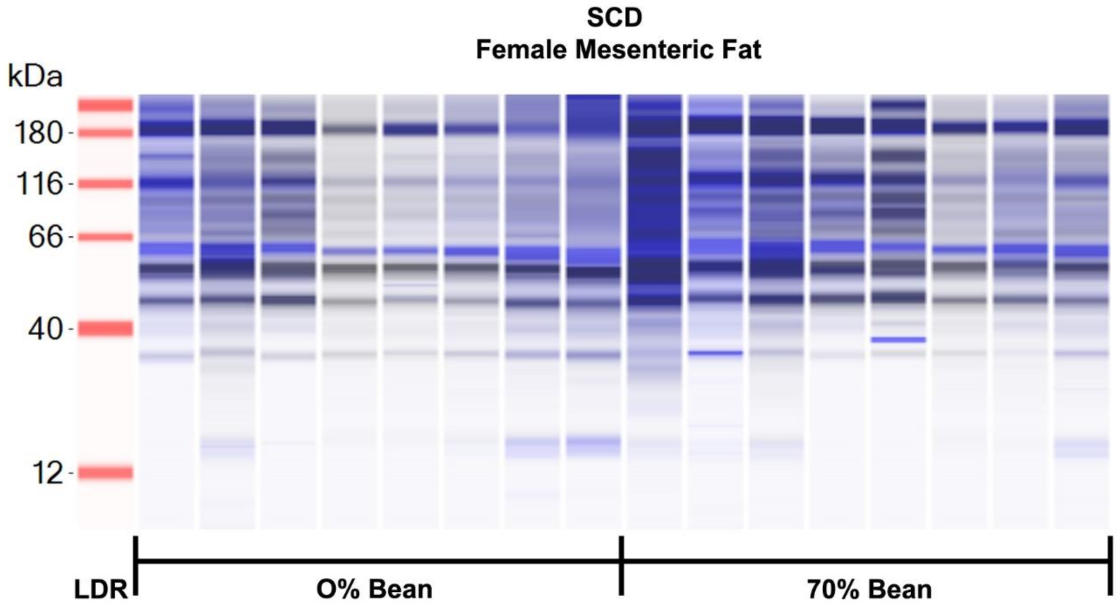

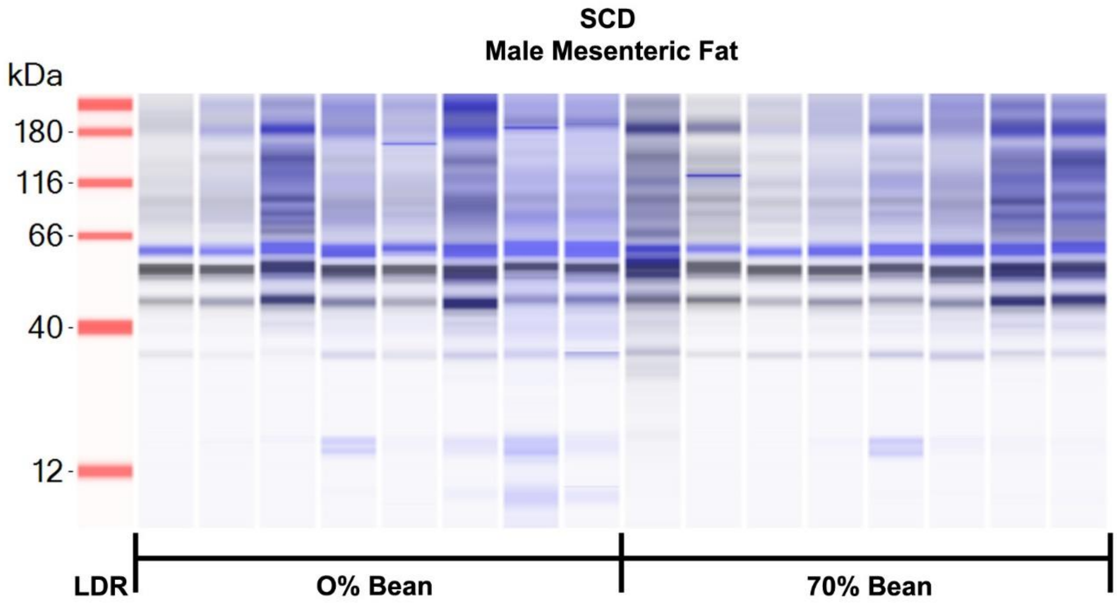

Figure S3 legend. The JESS Western blot analysis system generates electropherograms that are digitally evaluated. The software allows the depiction of electropherograms as Western blot images. The indicated proteins appear as dark bands. Signal normalization is via quantifying the total amount of protein loaded onto a capillary shown in blue.
